# Experimental dataset of fluid flow and heat transfer in a shallow packed bed at low Reynolds numbers

**DOI:** 10.1016/j.dib.2025.111743

**Published:** 2025-05-31

**Authors:** Anika Weber, Sebastian Schürg, Timo Roeder, Johannes Grobbel, Martina Neises-von Puttkamer, Christian Sattler

**Affiliations:** a German Aerospace Center (DLR), Institute of Future Fuels, Im Langenbroich 13, 52428 Jülich, Germany; b German Aerospace Center (DLR), Institute of Future Fuels, Linder Höhe, 51147, Cologne, Germany; c RWTH Aachen University, Chair for Solar Fuel Production, Templergraben 55, Aachen, Germany

**Keywords:** Particle bed, Plug flow, Particle-fluid heat transfer, Low aspect ratio, Thermal energy storage, Validation data, Emissivity, Infrared

## Abstract

This article presents an experimental campaign on the transient behaviour of a packed bed, which is filled with spherical particles and is subjected to heating and cooling with air as the heat transfer fluid. The investigation focuses on a shallow bed with a diameter twice its height, a geometry alike to cost effective thermal energy storage systems, where the bed-to-particle diameter ratio is typically also large.

The packed bed is heated from ambient temperature to temperatures up to 773K using hot air from an air heater. Then, when a specified shutdown criterion is reached, the heater is turned off and the bed is cooled by air with nearly ambient temperature. Following this procedure, 9 experiments have been conducted. The fluid velocity into the packed bed was conditioned to be almost homogeneous, which is achieved by wire screens and checked for in detailed flow pretests.

For each of the 9 experiments, the generated data consists of measurements of various temperatures, pressures, ambient conditions and air mass flow, all collected about 60 times per second during the whole experiment, i.e. from the beginning of heating to the end of cooling, which takes up to 300 minutes. Operational data of heater and blower are saved as well. Data is presented in form of tables (CSV,ASCII).

To collect the data, extensive instrumentation is employed, including more than 40 thermocouples, which are placed within the packed bed at multiple circumferential, radial, and axial positions and within the insulation, as well as above and below the bed. Pressure transducers are installed up- and downstream of the packed bed. A long-range infrared camera has an unobstructed view to the bed surface, enabling the investigation of average bed surface temperature, and thus, adding value to the point measurement data of the thermocouples.

Additionally, the publication includes data on material properties of insulation and particles. For example, bulk density was measured by pouring particles in a defined volume and measuring their mass. Particle size distribution and sphericity is evaluated based on image analysis and 3614 random selected particles. Here, raw data of the evaluation as well as the respective images can be found in the accompanying data set. Regarding other data such as specific heat capacity and thermal conductivity, relevant information is presented in form of equations and values. Particle emissivity is measured in-house with a simplified experimental setup, that is adaptable to other materials.

The detailed presentation of experimental methodology, material properties, flow pretests with and without fluid homogenization screens installed, and exemplary result description, ensures the results are suitable for validating advanced models such as detailed simulations based on Computational Fluid Dynamics (CFD) coupled with the Discrete Element Method (DEM) of shallow packed bed systems. Additionally, information about fluid homogenization with metal screens has the potential to support researchers in the design of similar test rigs.

## Graphical abstract

**Figure:**
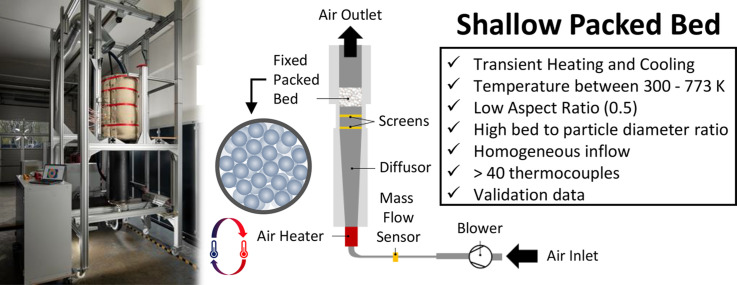

Specifications Table

**Table:** 

Subject	Engineering & Materials science
Specific subject area	Experimental data of transient cooling and heating behaviour of a shallow packed bed system with air as heat transfer media.
Type of data	Raw and processed (image evaluation) data presented in tables (CSV with comma as separator, ASC, XLSX), graphs (JPG, PNG, Python scripts), and images (IRBIS).
Data collection	Data • of in-house calibrated type N (500mm length) thermocouples (class 2) with 1mm diameter and a 5500mm long connection of type “compensating”, • of a type K thermocouple installed to read the heater temperature, • of a differential pressure sensor (IDS2, ICS Schneider Messtechnik GmbH), • of a volumetric flow sensor (IVA 520, ICS Schneider Messtechnik GmbH), • of an ambient condition sensor (S-THP-01a, Antratek Electronics Deutschland) and • of an air blower and air heater
	were collected using voltage and current input modules (Advantech ADAM 5018 series). An in-house LabVIEW program converts the electric signals to temperature, pressure or other data according to the datasheets of the devices. The data of the long range infrared camera (VarioCAM HD head 800 research, Infratec GmbH) has been collected by the Software IRBIS3 professional. Infrared data has been analysed using the same software. More specifically, packed bed average, maximum and minimum temperature were evaluated for each frame taken, and are saved in the respective experimental folder. Fluid velocity before fluid inlet into the packed bed was measured once during cold tests using the “TSI8455” velocity transmitter by Driesen+Kern. Values are read from the screen and noted manually. Results can be found in a Python file, which plots the same values. Raw data is presented in the Mendeley repository and data normalization or filtering has not been implemented. More information on the measurement system can be found in section 2 “Measurement system” of the article.
Data source location	Measurements took place at the German Aerospace Center, Institute of Future Fuels in Juelich, Germany.
Data accessibility	Repository name: Mendeley Data Data identification number: doi:10.17632/3pp86gdvh4.2 Direct URL to data: https://data.mendeley.com/datasets/3pp86gdvh4/2
Related research article	None.

## 1 Value of the Data

• By today, many researchers investigate the performance (economic or technical) of their engineered thermal energy storages or reactor concepts using detailed simulations and numerical models. In some cases, even safety related decisions may be based on such simulations. However, these simulations need proper validation, for which extensive information about the experimental conditions, materials and raw measurement data is necessary. Unfortunately, many available studies lack essential information and hence cannot provide a sufficiently comprehensive dataset for validation. Thus, this dataset aims at closing this gap by providing detailed information about the specific design of the test rig, material properties, sensors and devices as well as measurement procedures, making boundary conditions transparent. As the raw data published alongside with this article is presented in form of tables with timestamps and not only in figures, as in most available articles with similar experiments, the data can be easily utilized for a direct comparison of simulation results to experimental data.
• Furthermore, the present dataset can help to understand flow maldistribution, which might be present in shallow packed beds due to limited homogenization or flow straightening, especially during cooling situations. A special case with inhomogeneous inflow into a packed bed has been tested specifically for this reason.
• Available packed bed experiments in the literature focus mostly on
  ○ beds with a higher aspect ratio, i.e. $AR=h_{\text{bed}}/d_{\text{bed}}\geq 1$ with bed height $h_{\text{bed}}$ and bed diameter $d_{\text{bed}}$;
  ○ beds with low bed diameter $d_{\text{bed}}$ to particle diameter $d_{p}$ ratio, i.e. $BPR=d_{\text{bed}}/d_{p}<30$;
  ○ ambient temperature conditions or low temperature cases below 400K with some including only the heating of a packed bed or few higher temperature cases, i.e. above 500K, which often lack information of air inflow distribution;
  ○ investigation of Nusselt number, reaction extent or fluid velocity. Detailed information about the test rig design and single temperature values are seldom sufficiently presented.
• As a conclusion, the dataset presented in this study adds to the available literature data by going beyond typical values. Precisely speaking, a lower bed aspect ratio, a higher bed to particle diameter ratio, higher temperatures and background information extends available studies, and transparent boundary conditions and experimental design allows the usage of the experimental data for validation purposes.

## 2 Background

Flow maldistribution can cause severe technical, safety related or economic difficulties (low efficiencies) in moving or fixed particle and catalytic reactors, industrial furnaces, heat exchangers or TES [1]. Thus, these systems are nowadays often evaluated and designed using CFD-DEM simulations. The primary objective of this dataset is the use for validation of these simulations. Many experiments exist for deeper packed beds, but shallow packed beds may suffer from limited homogenization and flow straightening [[2], [3], [4]] as particles act as flow straightener. Hence, flow may be different compared to those beds of greater aspect (height to diameter) ratio. Nonetheless, due to cost optima, bed aspect ratios less than 1 are interesting for applications such as thermal energy storage (TES) systems [5].

**Fig. 1:**
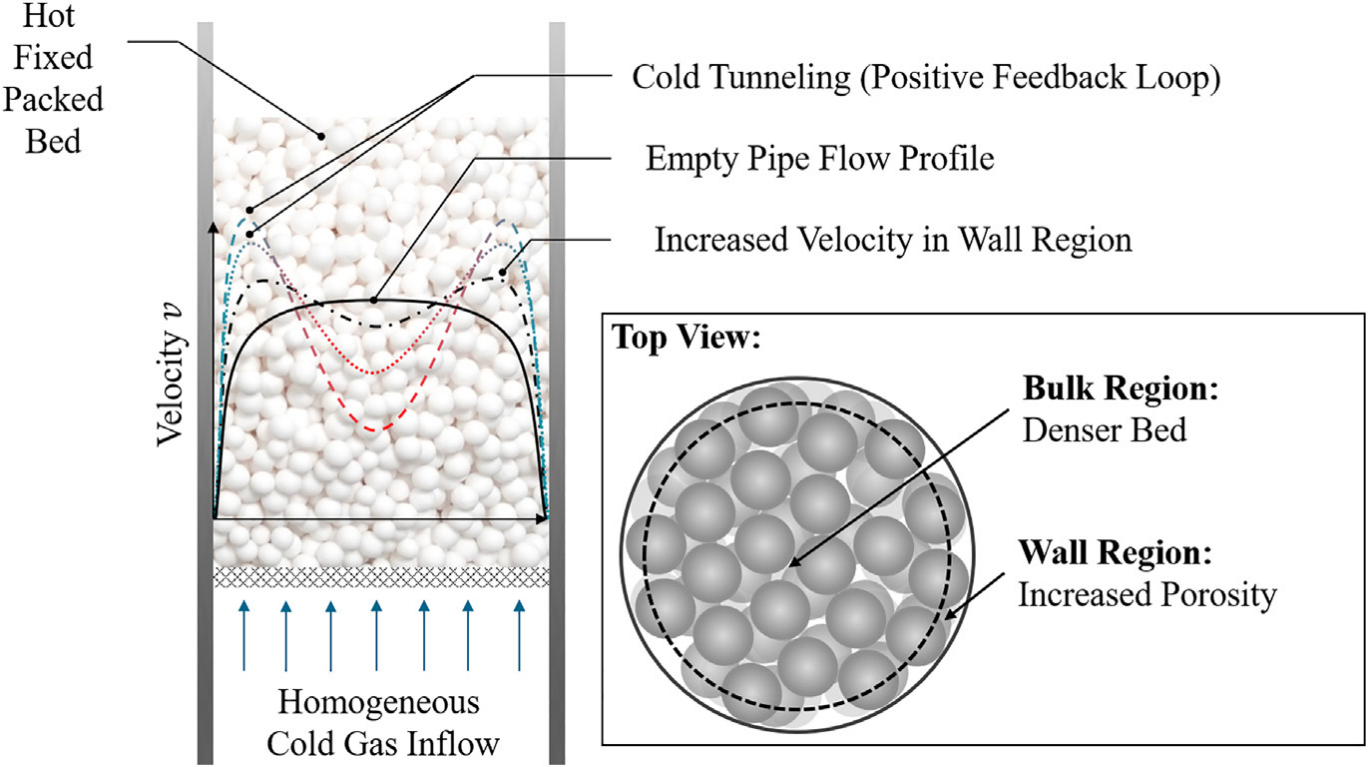
Schematic illustration of channelling in packed beds. The wall region shows an increased porosity, leading to a higher gas velocity.

In addition, the flow profile could differ between heating and cooling cases: Due to an increased porosity in wall regions or due to uneven filling of the packed bed, the fluid experiences less resistance in some bed regions, leading to an increased local fluid velocity, see Fig. 1 for the illustration in case of an increased porosity in wall regions. Reasoned in this is an effect called thermal channeling [6], cold tunneling [7] or finger penetration [8], which is only relevant in the cooling of a packed bed due to combination of mass flows, heat capacity flow rates, density and viscosity changes, which might result in a self-reinforcing effect. Even though this effect is perhaps more significant for a bed to particle diameter ratio less than 30 [9,10], cooling experiments are of great interest for validation of CFD-DEM simulations.

## 3 Data Description

This data set consists of 9 packed bed experiments categorized according to the volumetric flow rate and inflow situation. Selected tests have been repeated two times with similar conditions to allow for a measurement uncertainty analysis.

For each experiment, measurement data is grouped into folders in an accessible repository (https://data.mendeley.com/datasets/3pp86gdvh4/2 with doi:10.17632/3pp86gdvh4.2 [16]).

The following tree diagram shows the data available in the MENDELEY repository. Note that for visualization purposes, low flow and high flow tests have been grouped, and, if applicable, variables (x,y,z) are inserted for a concise representation of the directories:



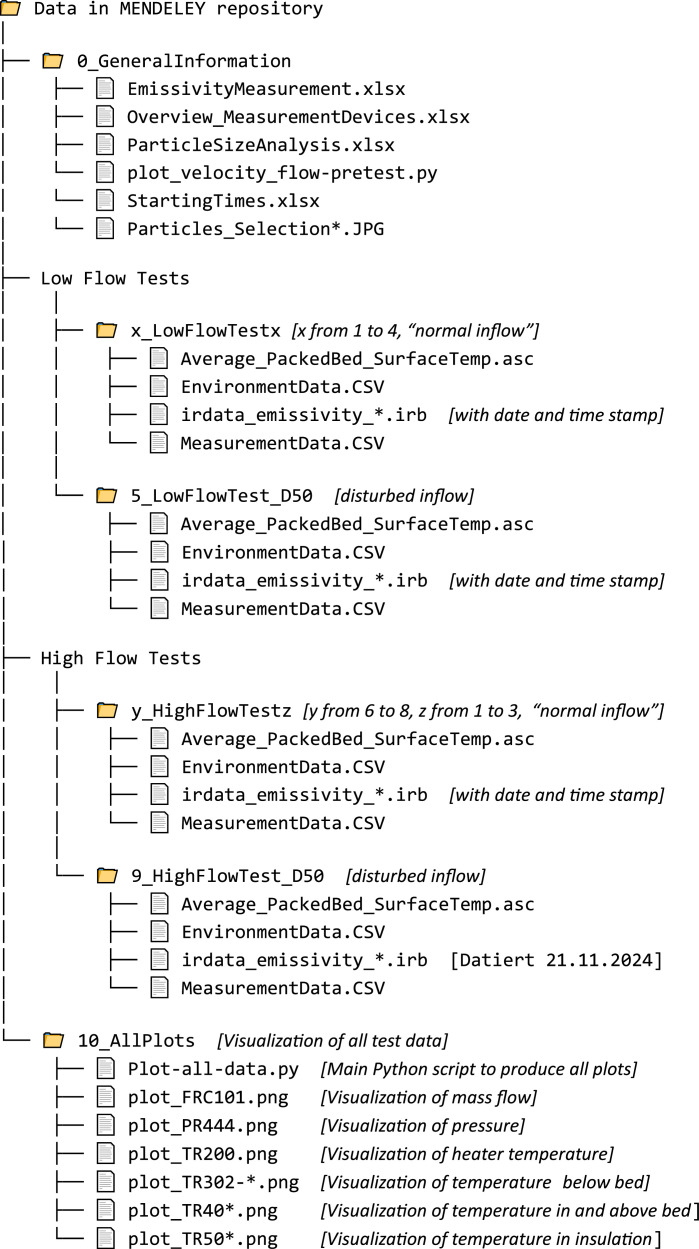



Table 1 describes the content of the files found in the data tree and in the actual Mendeley repository.

**Table 1:** Description of data set files.

File title	Content
EmissivityMeasurement.xlsx	This file provides background information to 9 emissivity experiments that have been conducted as described in section “Emissivity” of the manuscript. Background information is for example the date, on which the experiment has been conducted, the total duration, in which the furnace was heated, the temperature reading of the thermocouple placed in the packed particle bed as well as information necessary for the evaluation of emissivity. Precisely speaking, latter information contains information about the gap temperature, the amount of selected particles, their temperature correction after emissivity correction and the thereby evaluated emissivity. The relative error of the average value of the emissivity (standard deviation devided by average emissivity of particles) is given as well as an error estimation based on a furnace overshoot, which is also graphically explained below in the same .xlsx sheet. Also presented is an exemplary heating curve of the furnace with markers on special events, i.e. marking the two heating phases, the addition of a lid and the onset of the measurement. Note that the .xlsx file does not only contain single values, but also formulas for calculation.
Overview_MeasurementDevices.xlsx	In this file, the measurement devices are described according to their label in the “MeasurementData.CSV”, their location in the experimental system and the functionality, which is either a differential pressure reading, a temperature measurement, device control or measurement of mass flow. The device types (type K or type N thermocouple, device names) as well as the ranges are presented. Importantly, the unit is given as in the “MeasurementData.CSV” file.
ParticleSizeAnalysis.xlsx	The file “ParticleSizeAnalysis.xlsx” contains the result of an image analysis from the software ImageJ of a random selection of 3614 particles. The measurement procedure is explained in the subsection “Particle Properties - Size” in the manuscript. In the file, each particle has an assigned number, an area, a perimeter and a Feret diameter as well as Feret angle. From the area, the area-based diameter is calculated. Based on perimeter and area-based diameter, the circularity is calculated. Error analysis is conducted for the area-based diameter and the circularity, i.e. the arithmetic mean and the standard deviation of a single measurement and of the average are calculated. The median is presented as well. Other diameter definitions, such as Sauter diameter or DeBroukere diameter are calculated. Finally, a histogram for diameter and circularity is shown. Alike to the file “EmissivityMeasurement.xlsx”, the file “ParticleSizeAnalysis.xlsx” does not only contain values, but also the formulas for their calculation.
plot_velocity_flow-pretest.py	The Python script “plot_velocity_flow-pretest.py” contains raw measurement data and the error calculation. It is mainly used for plotting fluid velocities, which were measured once with cold flow present below the packed bed, i.e. where the fluid enters the packed bed. Detailed information about the measurement procedure and a result description can be found in section “Flow Pretests” in the manuscript.
StartingTimes.xlsx	In this file, the start time of the camera and the start time of the LabVIEW program are noted for each single experiment. Hence, it allows for a comparison between image files (“irdata_emissivity_*.irb”) and tabular data (e.g. “MeasurementData.CSV”), as image files and the tabular data are based on relative time since start.
Particles_Selection*.JPG	Both figures together show 3614 randomly selected particles (white) on a rough surface (black). A scale is shown as well. The figures were used in the Software ImageJ to evaluate particle size properties (diameter, sphericity, size histogram, etc.).
EnvironmentData.CSV	In this file, the Labview program saves the ambient condition data: the temperature (in°C), the humidity (in percent), the dew point (in°C), and the air pressure (in hPa) measured by the S-THP-01A sensor from the company Antratek Electronics Deutschland, see the measurement section of the manuscript. The data is saved regularly, about 60 times per second.
irdata_emissivity_*.irb	The .irb file contains the thermal image data of the long-range infrared camera “VarioCAM HD head 800 research” by the company Infratec. The file name contains further information about the sampling rate (for eample, Intervall60s meaning that every 60 seconds a picture is taken), and about the date and time the measurement took place. As .irb data is not a common file type, and hence not easily processed by everyone, the authors decided to include postprocessed information which has been evaluated by the Software IRBIS3 professional, see “Average_PackedBed_SurfaceTemp.asc”.
Average_PackedBed_SurfaceTemp.asc	The ASCII files contain information that is used for example in Fig. 28 of the manuscript. Precisely speaking, it shows the analyzed results of the Software IRBIS3 Professional: the name of the file and then for each figure taken the index, the average temperature of the packed bed, the maximum temperature of the packed bed, the minimum temperature of the packed bed and where maximum and minimum are located in the figure (pixel coordinates). The first line of the ASCII files tells the date when the results have been created.
MeasurementData.CSV	This file contains numeric, tabular like data of the mass flow sensor (Nm³/h), thermocouples (°C), pressure transducer (mbar) and the voltage and current setting of the blower (Altivar_AO) and heater (Heater_AO), respectively. Data has been saved by the in-house Labview program. Alike to “EnvironmentData.CSV”, data is saved about 60 times per second. Again, timestamps are relative to the start of the LabVIEW program.
Plot-all-data.py and plot_*.png	This python script generates .png files that can be found in the same folder. The purpose is to provide a quick overview of all data across all folders.

Table 2 presents a brief overview over the respective experiments in the respective folders.

**Table 2:** Short overview over experimental tests.

Folder	Folder Title	Parameter
1	Low Flow Test 1	Date: 23^rd^ October 2024 Heating Time: 210 min (Shutdown criteria: time) Cooling Flow Rate: 15 Nm³/h Inflow: Homogeneous inflow to the particles
2	Low Flow Test 2	Date: 30^th^ October 2024 Heating Time: 210 min (Shutdown criteria: time) Cooling Flow Rate: 15 Nm³/h Inflow: Homogeneous inflow to the particles
3	Low Flow Test 3	Date: 31^st^ October 2024 Heating Time: 210 min (Shutdown criteria: time) Cooling Flow Rate: 15 Nm³/h Inflow: Homogeneous inflow to the particles
4	Low Flow Test 4	Date: 14^th^ November 2024 Heating Time: ca. 83 min (Shutdown criteria: gradient) Cooling Flow Rate: 15 Nm³/h Inflow: Homogeneous inflow to the particles
5	Low Flow Test D 50 Inflow	Date: 20^th^ November 2024 Heating Time: ca. 82 min (Shutdown criteria: gradient) Cooling Flow Rate: 15 Nm³/h Inflow: A 50 mm diameter plate was placed below the particles.
6	High Flow Test 1	Date: 5^th^ December 2024 Heating Time: ca. 82 min (Shutdown criteria: gradient) Cooling Flow Rate: 38 Nm³/h Inflow: Homogeneous inflow to the particles.
7	High Flow Test 2	Date: 14^th^ January 2025 (morning) Heating Time: ca. 86 min (Shutdown criteria: gradient) Cooling Flow Rate: 38 Nm³/h Inflow: Homogeneous inflow to the particles.
8	High Flow Test 3	Date: 14^th^ January 2025 (afternoon) Heating Time: ca. 82 min (Shutdown criteria: gradient) Cooling Flow Rate: 38 Nm³/h Inflow: Homogeneous inflow to the particles.
9	High Flow Test D 50 Inflow	Date: 21^st^ November 2024 Heating Time: ca. 77 min (Shutdown criteria: gradient) Cooling Flow Rate: 38 Nm³/h Inflow: A 50 mm diameter plate was placed below the particles.

Within the following subsections, the data from the previously briefly described experiments is presented. Note that it might be of the readers interest to familiarize with the test stand first, which is presented in the section “Experimental Design, Materials and Methods”.

### 3.1 Repeatability experiments

To provide information about the measurement uncertainty, two repetitions have been performed for both low ($15\;Nm^{3}h^{-1}$) and high ($38\;Nm^{3}h^{-1}$) flow rates. In these repetitions, the heating time was kept approximately (see raw data) constant, neglecting the previously mentioned end of heating (gradient) criteria. The exact values can be found in Table 3. Within these repetition tests, the packed bed has been newly poured every time. Additionally, it should be noted that insulation and heater have been untouched between all experiments to ensure that conditions in all tests are truly comparable.

**Table 3:** Settings for repetition tests.

Component	Test with Lower Flow Rate	Test with Higher Flow Rate
Amount of tests performed	3	3
Heating mass flow	$38\;Nm^{3}h^{-1}$	$38\;Nm^{3}h^{-1}$
Heating time	210min	(82−86)min
Cooling mass flow	$15\;Nm^{3}h^{-1}$	$38\;Nm^{3}h^{-1}$

Different flow rates have been tested, which are summarized in the table below:

Standard deviations of the repetition tests are calculated using the “numpy.std” functionality in Python after shortening the data manually so that all data arrays have the same length. To shorten the data, each array contained the same number of rows for the heating phase and the same number of rows for the cooling phase, removing initial and final rows. The start of the heating can be seen in the data as the heater control switches from 4mA to 20mA. Along the same lines, the start of cooling can be seen from the switch back to 4mA. Note that these standard deviations are used for the plate case, in the following abbreviated with D50, as well. No extra repetition test has been performed.

### 3.2 Repeatability: low flow rate

**Fig. 2:**
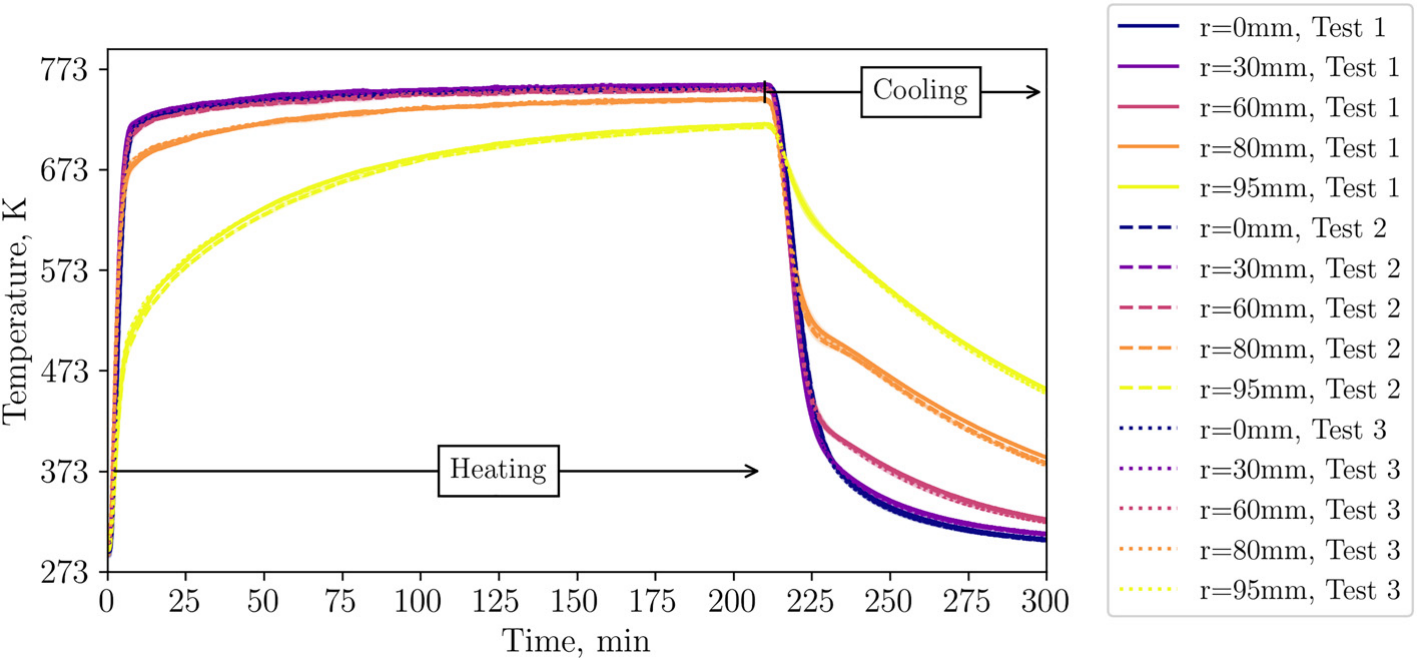
Temperatures in level 1 (tests from 23^rd^, 30^th^ and 31^st^ of October 2024).

Fig. 2 shows the level 1 TC data of all three repetition tests from the start of the heating until the end of the cooling phase. The cooling start can be clearly seen as a drop in the temperature level. The behavior between different tests is almost identical: During heating, TCs which are closer to the center (r=95mm) reach higher temperatures, while temperatures close to the wall, i.e. with a distance from center of 95mm show lower temperatures. During cooling, the situation switches and near wall TCs show higher temperatures due to the hot insulation and higher wall temperatures. TCs in layer 2 and 3 show a similar behaviour near the wall as those in level 1. Thus, they are not shown in this summary but data can be found in the data set.

**Fig. 3:**
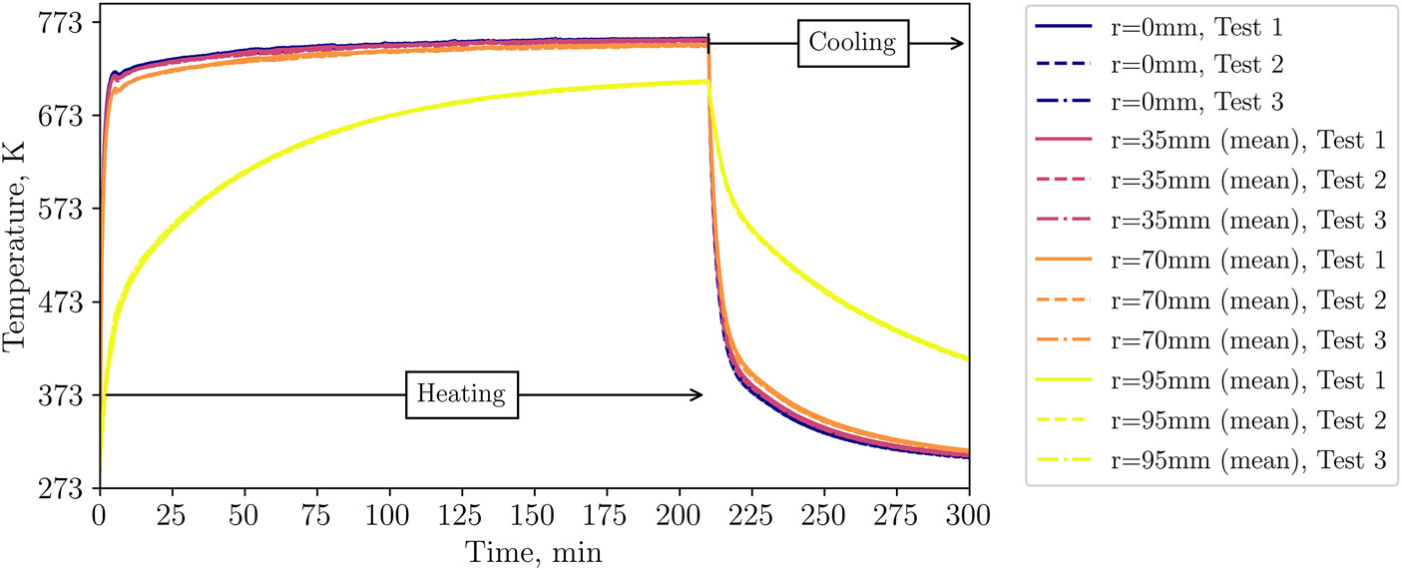
Thermocouple below bed during repetition tests (tests from 23^rd^, 30^th^ and 31^st^ of October 2024). In case that several thermocouples at one radial distance are installed, their average is displayed.

**Fig. 4:**
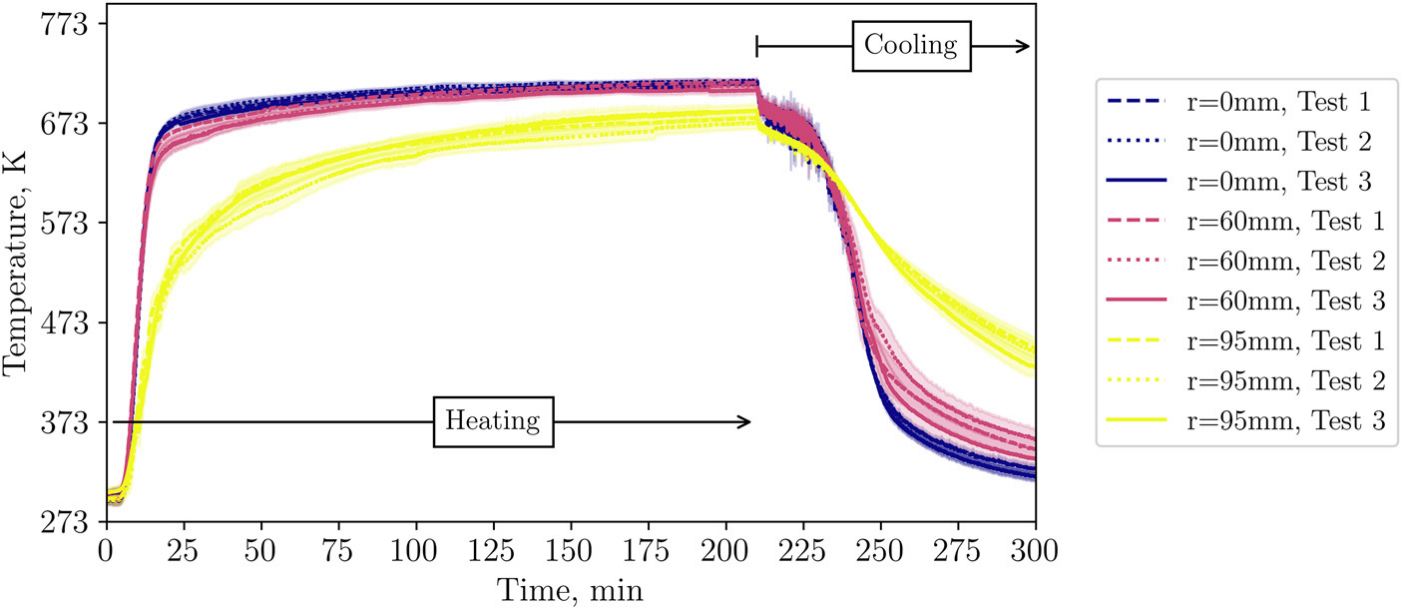
Air outlet temperatures during repetition tests (tests from 23^rd^, 30^th^ and 31^st^ of October 2024).

A similar behavior of near wall TCs is found for the TCs below the packed bed (Fig. 3) and for the air outlet temperature (Fig. 4).

**Fig. 5:**
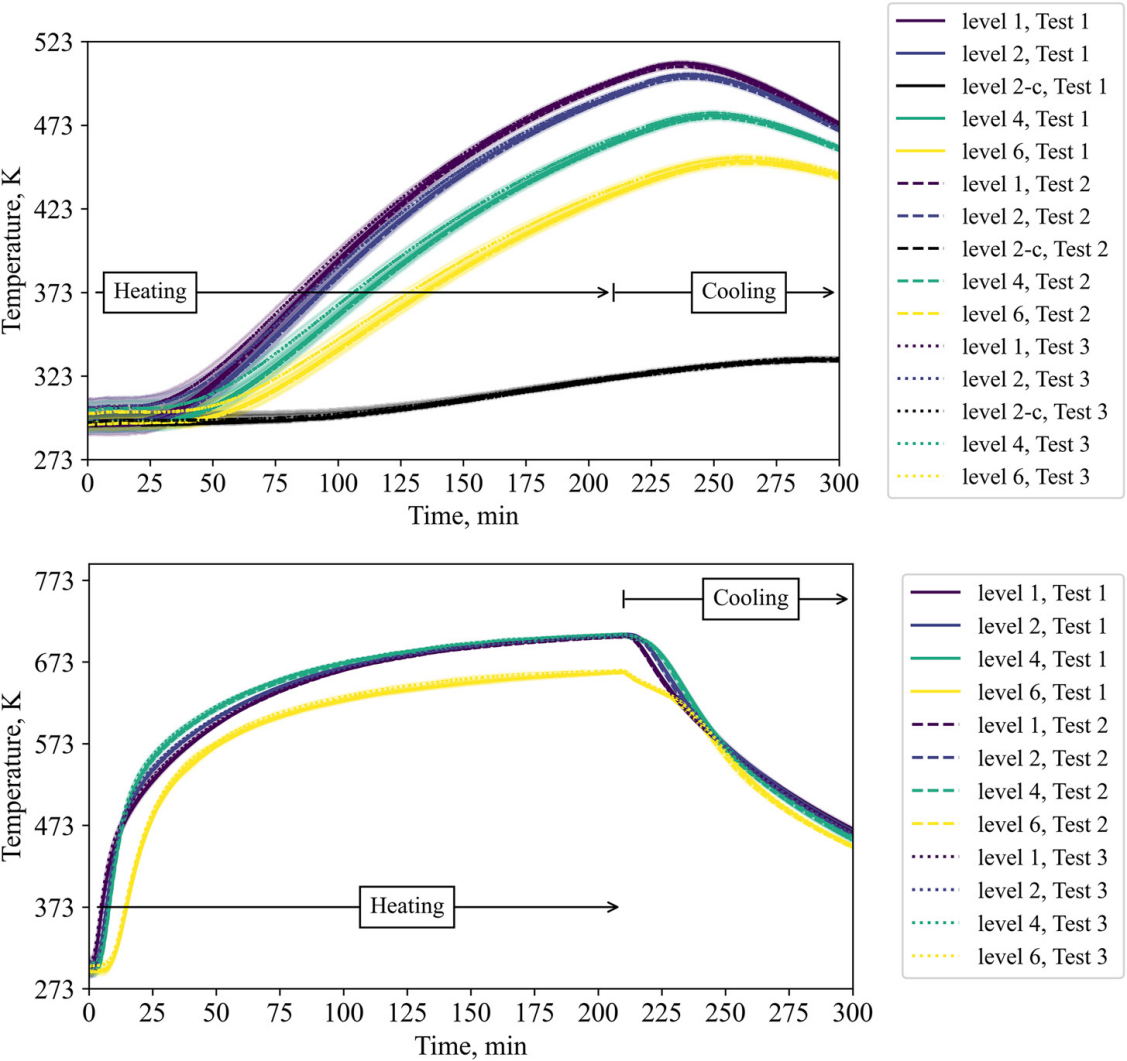
Temperatures in insulation (top) at 100 mm insertion depth and 25 mm insertion depth (“level 2-c”) and steel (bottom) during repetition tests (tests from 23^rd^, 30^th^ and 31^st^ of October 2024).

The temperature of the insulation and steel are shown in Fig. 5 for different levels. Naturally, the steel temperature response is faster. The steel temperature of level 6 shows an interesting temperature drop behavior: Directly at the first minutes after the start of cooling a slight drop is visible. This drop gets more pronounced after 240 minutes.

**Fig. 6:**
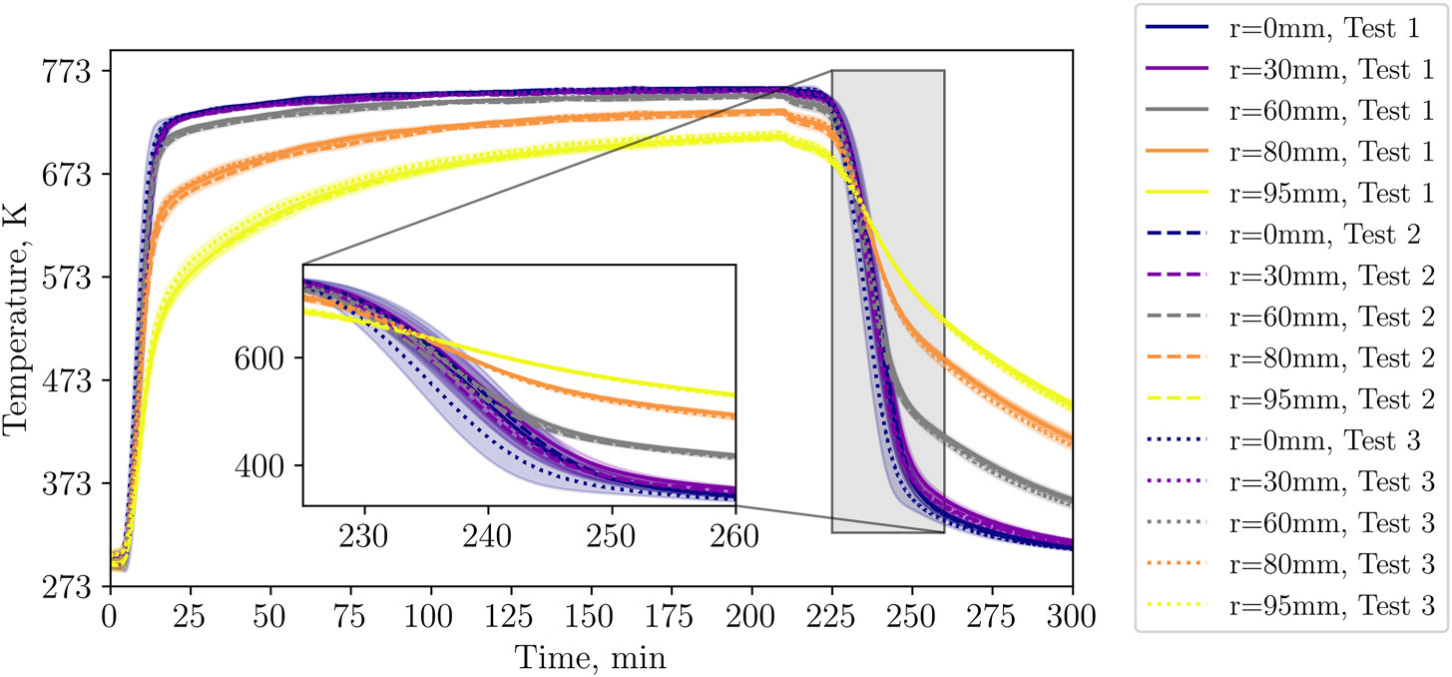
Temperatures in layer 4 during repetition tests (tests from 23^rd^, 30^th^ and 31^st^ of October 2024).

**Fig. 7:**
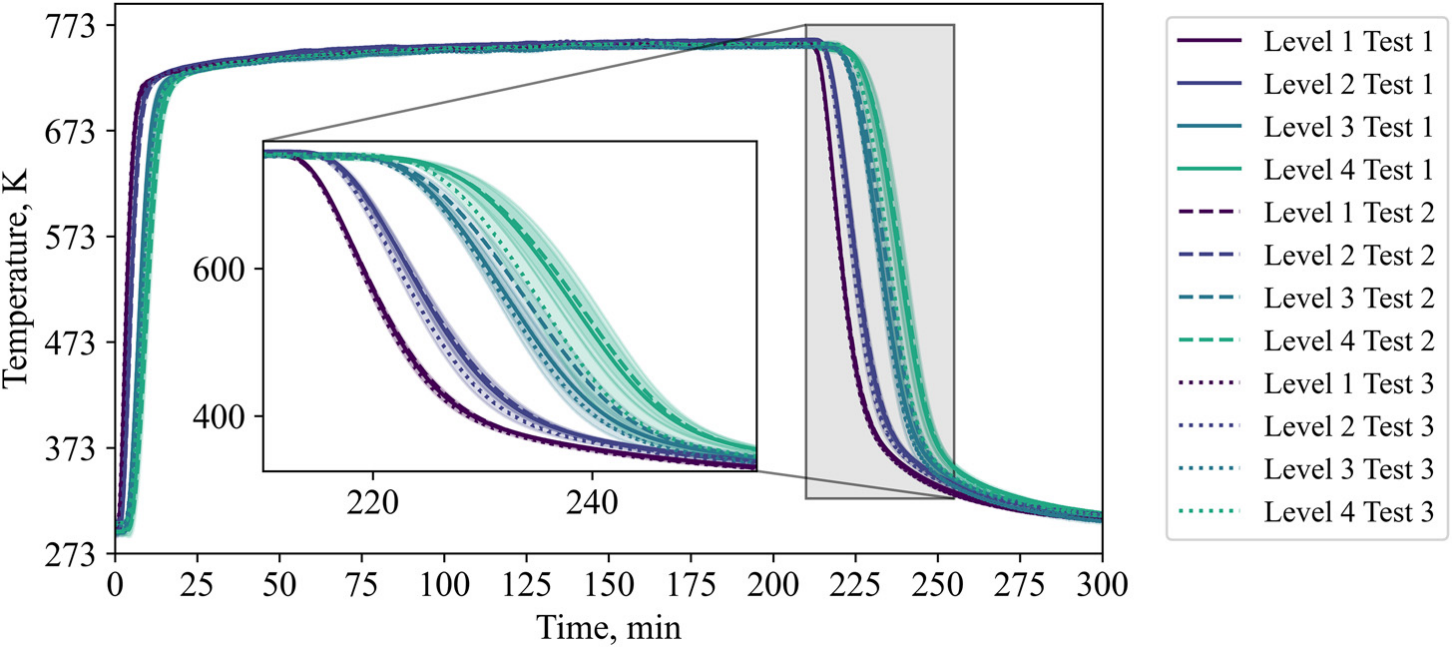
Central thermocouples within the bed during the repetition tests (tests from 23^rd^, 30^th^ and 31^st^ of October 2024).

Regarding standard deviations, the biggest difference is calculated for the TCs in layer 4, and here, especially for the center TCs. The callout in Fig. 6, Fig. 7 shows the time when the biggest differences occur. Standard deviations are shown as colored marked area. Note that standard deviations are shown in the previous plots as well. However, owing to their negligible magnitude, they are almost not visible (except of the TC measuring the air outlet temperature).

To compare different levels in one plot, the center thermocouples are shown in Fig. 7. A classic thermocline is visible with standard deviations between different tests increasing per layer towards the top of the bed.

**Fig. 8:**
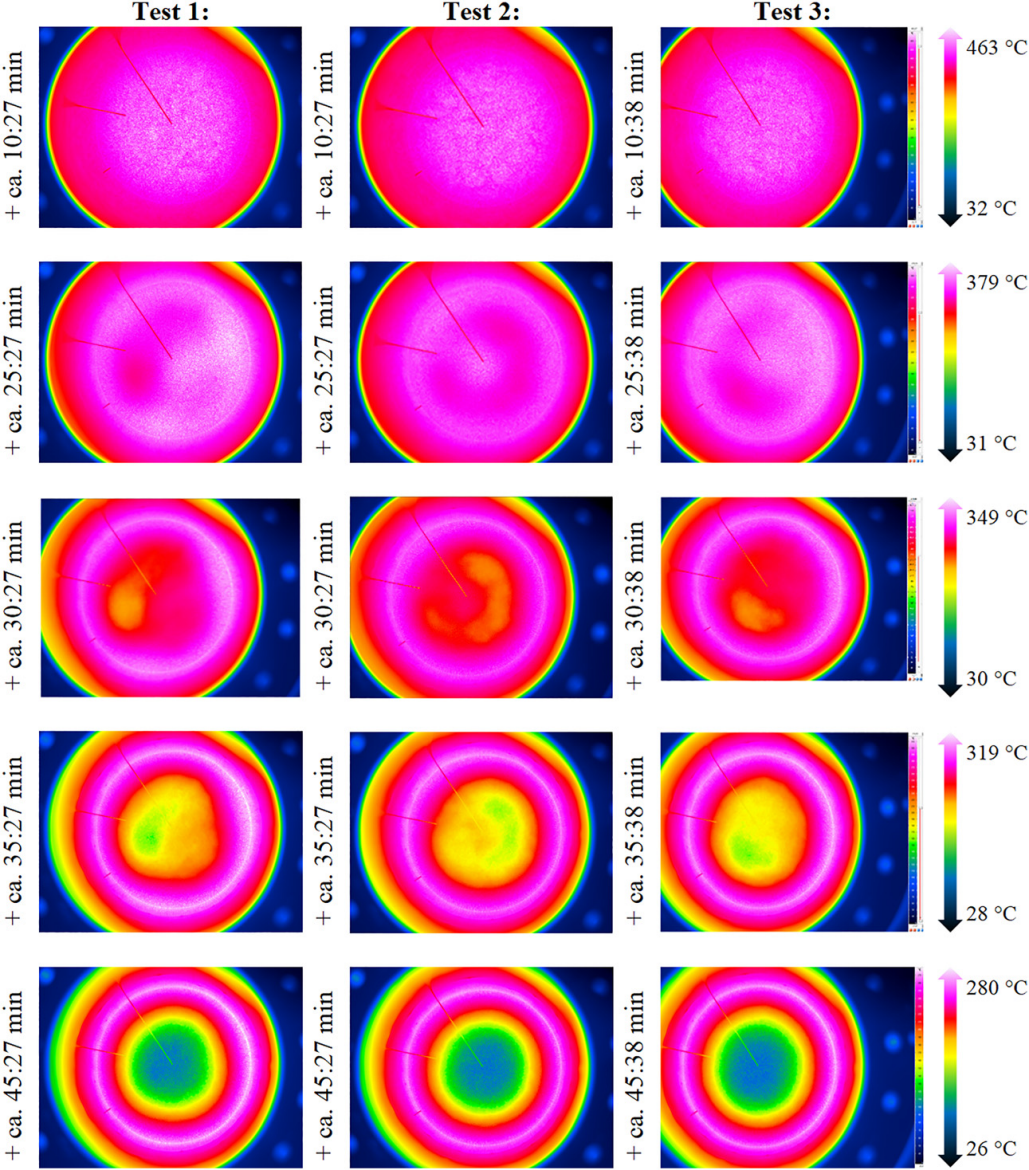
Comparison of IR data for repetition tests (tests from 23^rd^, 30^th^ and 31^st^ of October 2024).

As the TC within the bed and shortly below the bed surface deliver data at 5 specific locations only, an IR camera is used to evaluate the whole bed surface. Exemplary screenshots of the resulting IR measurements are shown in Fig. 8. In each row, the temperature range is identical. From the figure, one can see the development and vanishing of slight differences between each packed bed experiment, i.e. some areas are cooling faster than others.

**Fig. 9:**
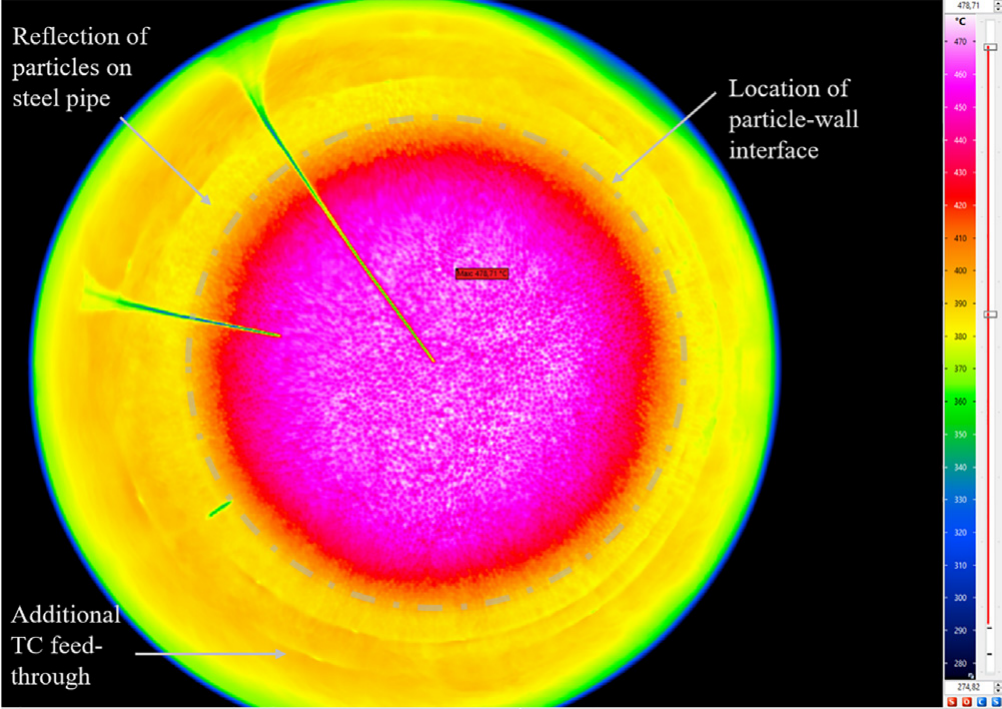
Selection of packed bed surface.

**Fig. 10:**
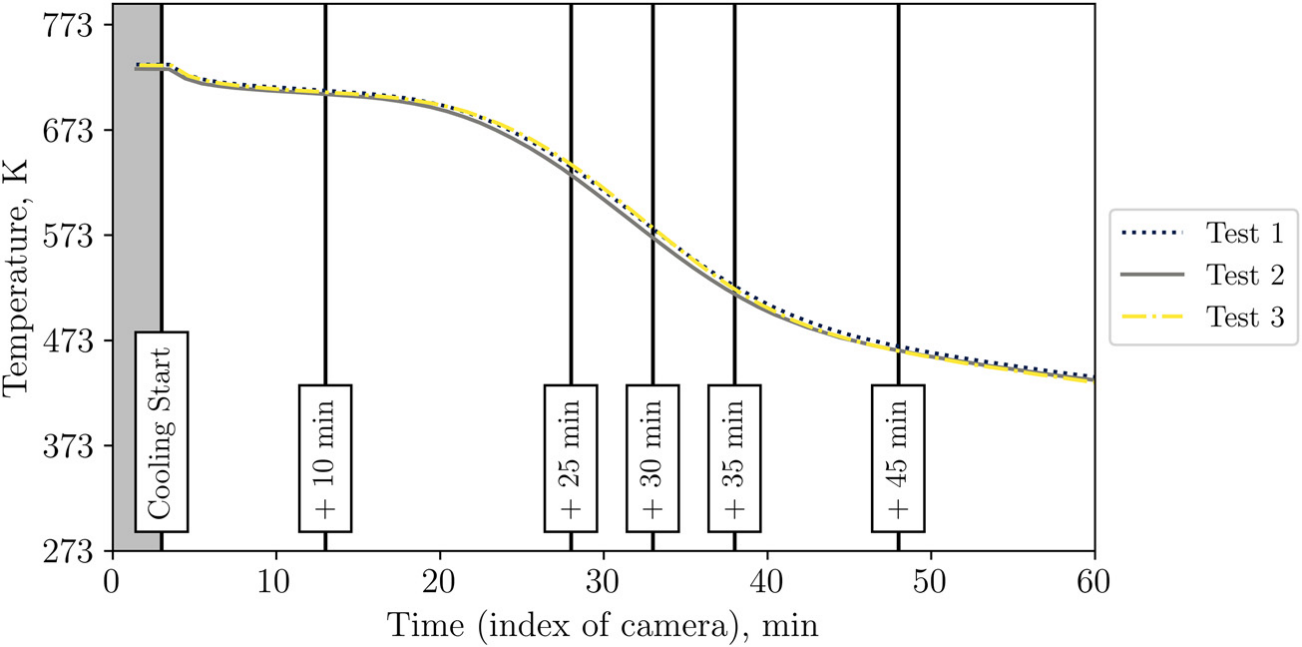
Average packed bed surface temperature vs. time during repetition tests (tests from 23^rd^, 30^th^ and 31^st^ of October 2024).

To determine whether the shown differences are significant or not, the bed surface is selected (circular area, see Fig. 9). For this surface, the temperature value is evaluated with an ε-correction of 0.965, which is the average value of the emissivity of the bed surface, see Fig. 31. Now, this corrected temperature value is evaluated for each IR picture (one per minute). In the raw camera data, the figures are saved as “index”. Thus, Fig. 10 shows not the time on the x-axis but a so called “index”. To increase understandability, the cooling start, and additional time stamps (compare to Fig. 8) are marked. On the y-axis, the averaged infrared temperature is shown. It can be seen that the average temperature is almost identical for each test.

**Fig. 11:**
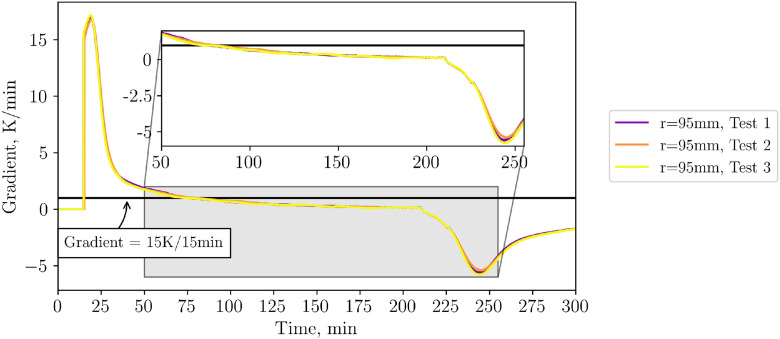
15-Minute gradient during repetition tests (tests from 23^rd^, 30^th^ and 31^st^ of October 2024). No standard deviation is calculated or shown in the figure.

Checking the reproducibility of the aforementioned end-of-heating / gradient criteria, Fig. 11 shows the 15-minute gradient for all three repeatability tests. From this it can be seen that the end of heating (gradient) criteria is reached at the same time for all three tests.

Note that the utilization of the end-of-heating gradient criteria in later experiments (see section “Comparison of Different Inflow Configurations”) leads to an earlier shutdown time (approximately a third of the repetition case heating time of 210min), which might result in a slightly more uneven temperature distribution. However, as the temporal changes are already small, no big influence is expected. This assumption is supported by the fact that the measurement errors for the high flow case with shorter and inequal heating time are in the same order of magnitude.

**Fig. 12:**
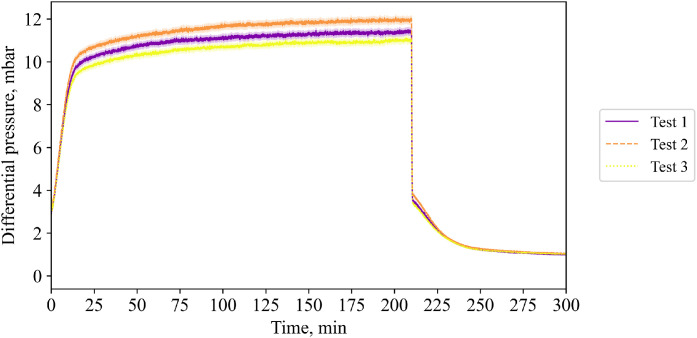
Differential pressure (tests from 23^rd^, 30^th^ and 31^st^ of October 2024) with accuracy of the measurement. No standard deviation is calculated or shown for this figure.

To complete the most relevant data description of the low flow case, Fig. 12 shows the differential pressure for the three tests.

Finally, it should be mentioned that the heating element was replaced due to a component failure: By changing the heating element, the channels (see Fig. 2d) of the heater changed their position so that the TC, which was previously exactly above a channel, was no longer aligned above this channel. This would have led to incorrect measurement results. The position of the TC was therefore changed so that it was once again above a channel. Due to this change, the temperature below the packed bed changed from 748K for the old placement to 763K for the new placement while the temperature reading of the heater control TC (TR200) showed 823K for both cases. To account for this deviation, all low flow repetition tests (data in folder 1, 2, and 3) were performed with the same heating element, and all other subsequent experiments were performed with a different one.

### 3.3 Repeatability: high flow rate

**Fig. 13:**
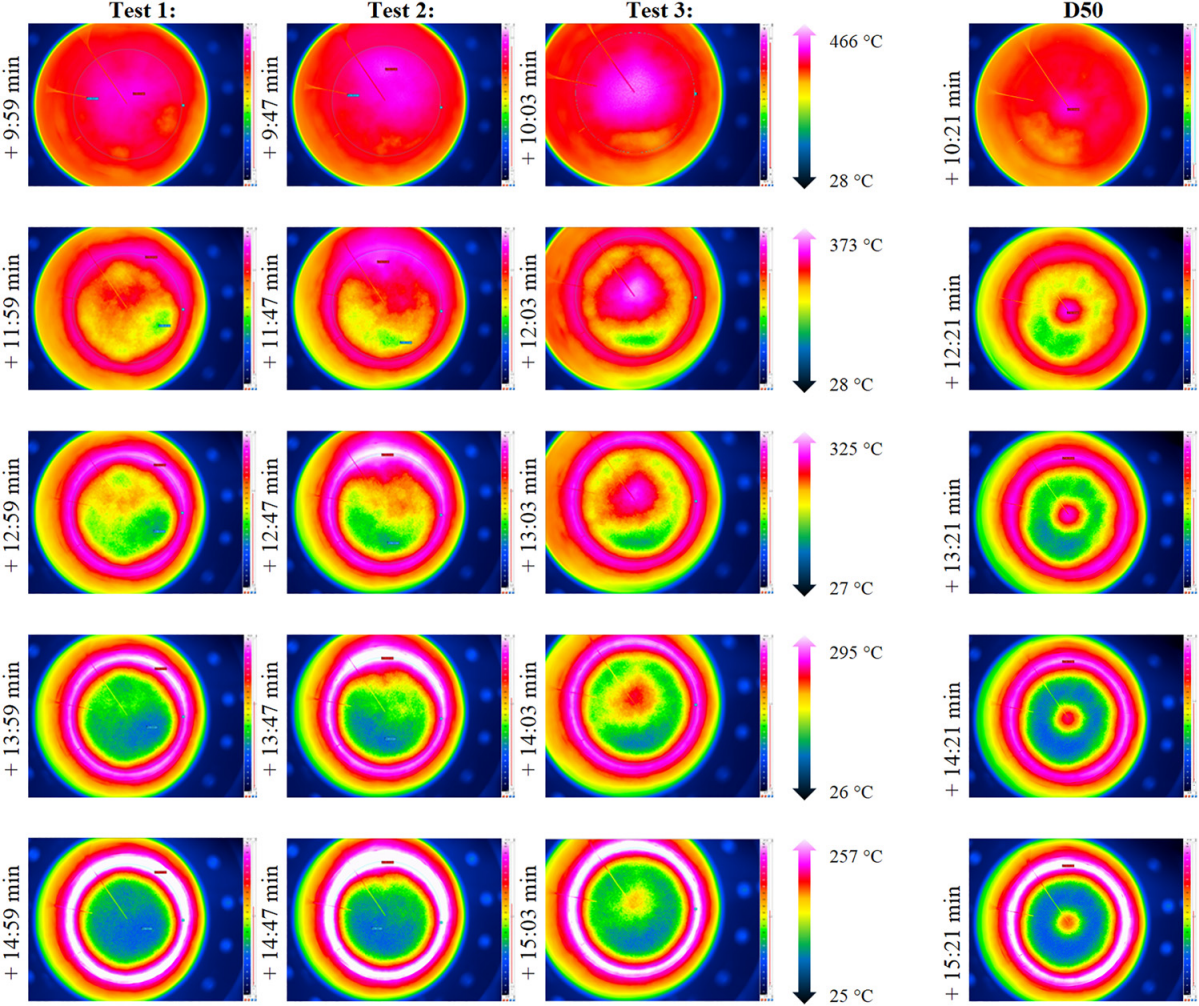
IR data for the repetition tests with a higher volumetric flow rate (tests from 21^st^ of November, 5^th^ December, and 14^th^ of January).

As the general TC behavior is similar to the low flow case and as the data can be found in the data set, the authors report only on the IR image data in this section, because of the complexity of processing of this data. Fig. 13 shows exemplary images with the respected time in relation to the start of cooling phase for the three repetition tests and, for comparison, the case with the non-homogeneous inflow due to a 50mm diameter plate placed below, which is not part of the repetition tests.

**Fig. 14:**
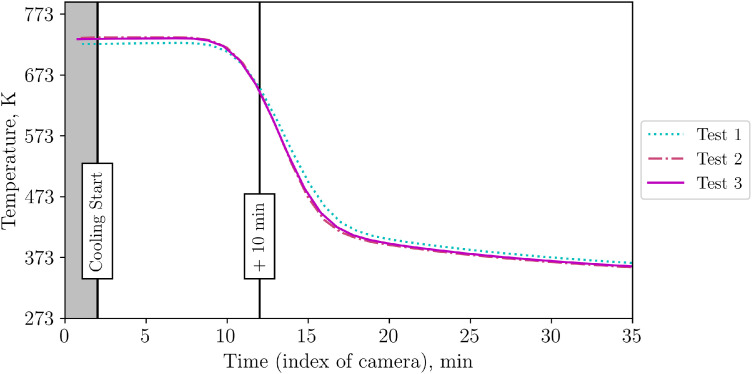
Average top surface temperature of packed bed during high flow repetition tests (tests from 5^th^ December, and 14^th^ of January.

Again, to distinguish between visible and impactful differences in Test 1, 2 and 3, the temperature on the top surface of the packed bed is averaged, see Fig. 14. It can be seen that all repetition tests show a similar course of average top surface temperature.

### 3.4 Comparison of different inflow configurations

**Fig. 15:**
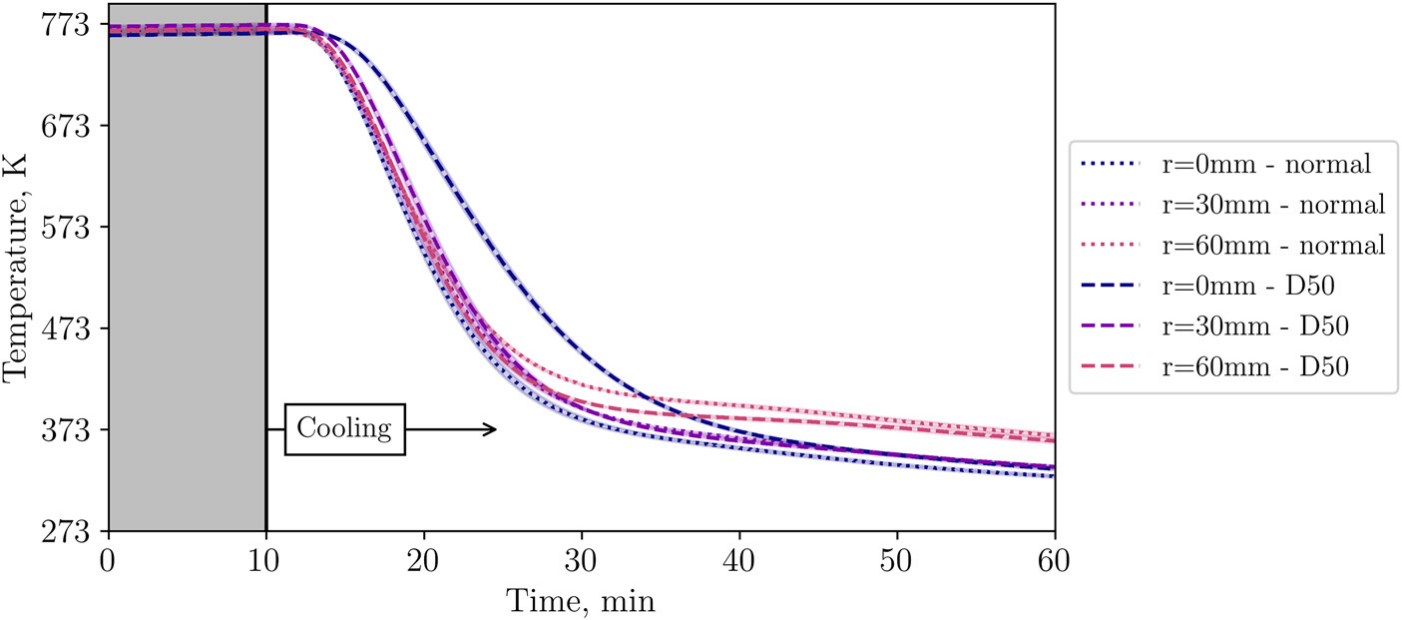
Comparison of temperatures in level 1 for two different inflow configurations at low volumetric flow rate (tests from 14^th^ and 20^th^ November).

Having executed three repetition tests for each of the high and low flow cases, different inflow configurations are now to be compared. To be clear, this is a comparison between one case with homogeneous inflow into the bed (“normal”) and another with inhomogeneous inflow due to a plate placed directly below the particles (“D50”). Fig. 15 shows the temperature in the first level of the packed bed for both cases at low volumetric flow rate. The standard deviation is assumed to be the same as in the respective low- or high flow repetition experiments without the plate as an obstacle.

Fig. 15 shows that all TCs in the first layer with well distributed inflow cool similarly fast. However, with the plate being installed, the center TC shows a significant delay. As stated before, the plate has a diameter of 50mm. Comparing the TC placed 30mm off center, both cases (with and without the plate) show similar values.

**Fig. 16:**
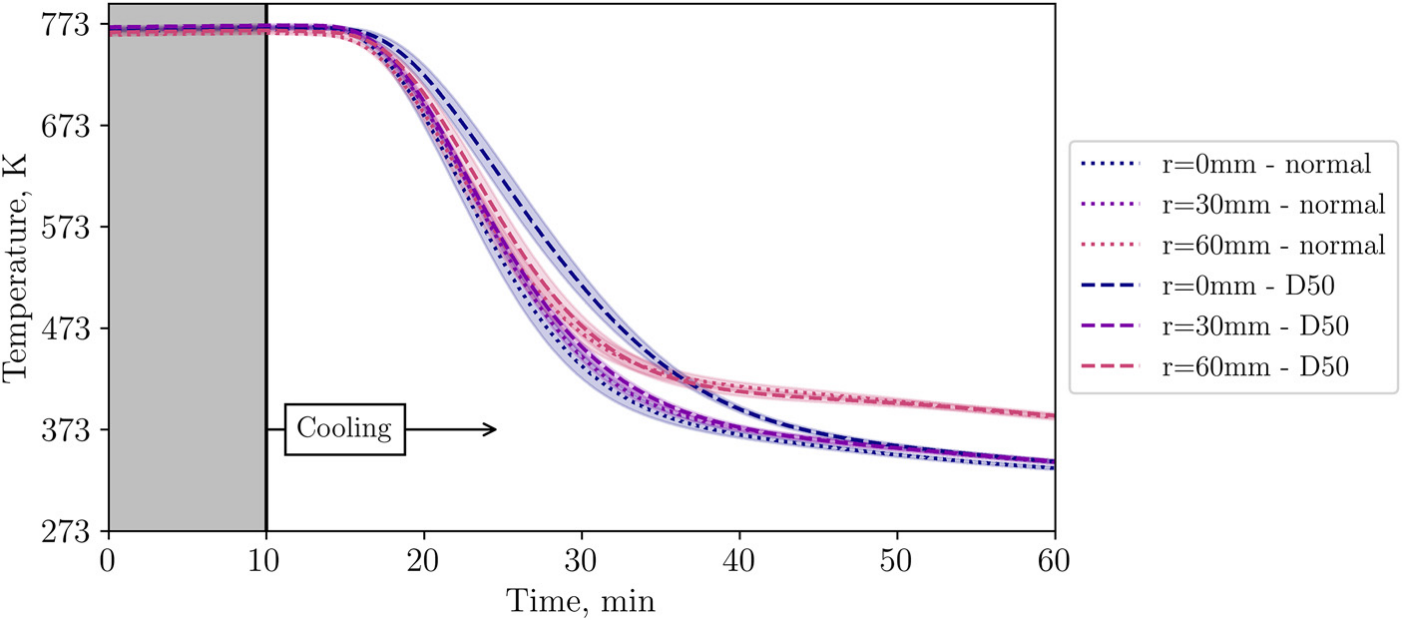
Comparison of temperatures in level 2 for two different inflow flow configurations at low volumetric flow rate (tests from 14^th^ and 20^th^ November).

**Fig. 17:**
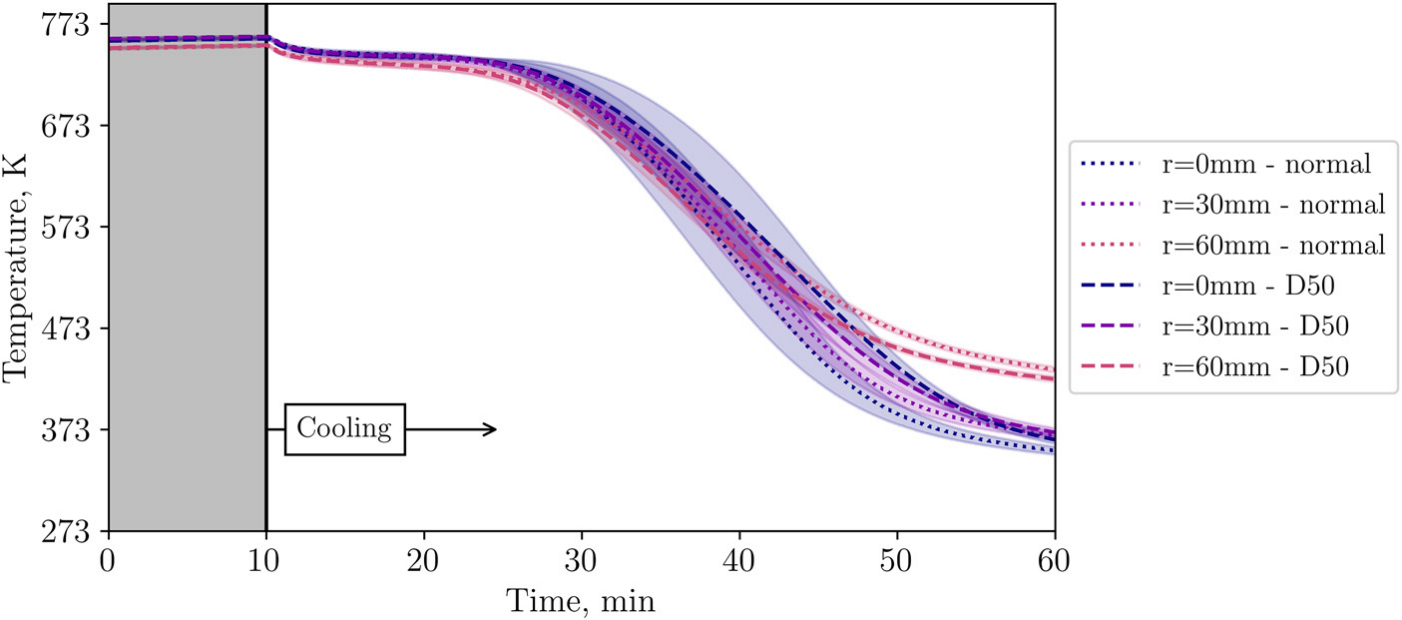
Comparison of temperatures in level 4 for two different inflow configurations at low volumetric flow rate (tests from 14^th^ and 20^th^ November).

To investigate the perturbation length of the disturbed inflow, Fig. 16, Fig. 17 show the succeeding levels 2 and 4.

**Fig. 18:**
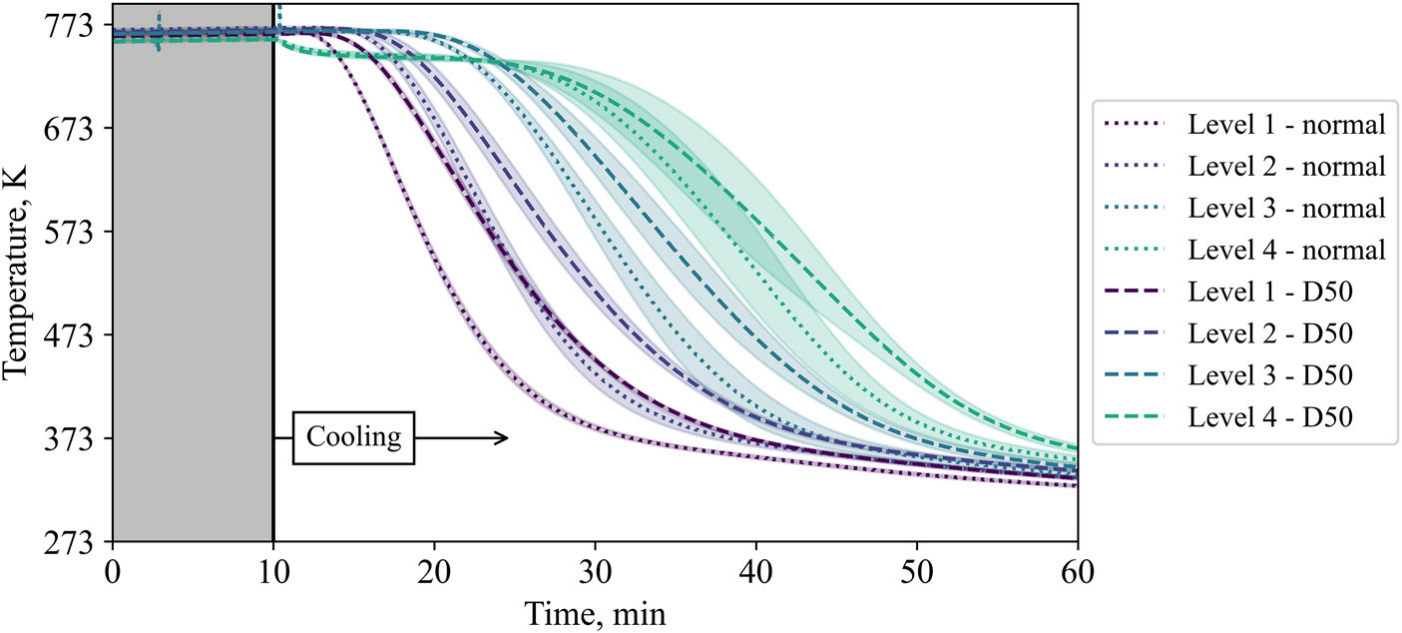
Comparison of temperatures in the center of the packed bed for two different inflow configurations at low volumetric flow rate (tests from 14^th^ and 20^th^ November).

Fig. 18 compares the central TC in the bed for different inflow configurations. Again, it can be clearly seen that the central TC shows more inertial behaviour when the plate is installed compared to the unobstructed inflow.

**Fig. 19:**
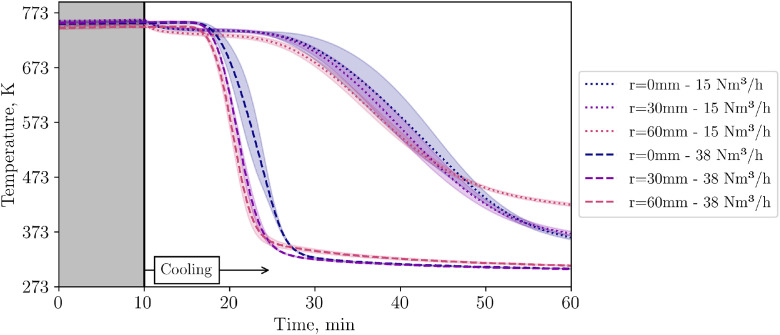
Comparison of temperatures in level 4 for two different flow rates with a 50mm diameter plate in the center below the particles (tests from 20^th^ and 21^st^ November).

So far, the results presented in this subchapter are for the low flow case. Now, in Fig. 19, TCs in level 4 are compared for different flow rates when a plate is present as an obstacle for the incoming flow.

**Fig. 20:**
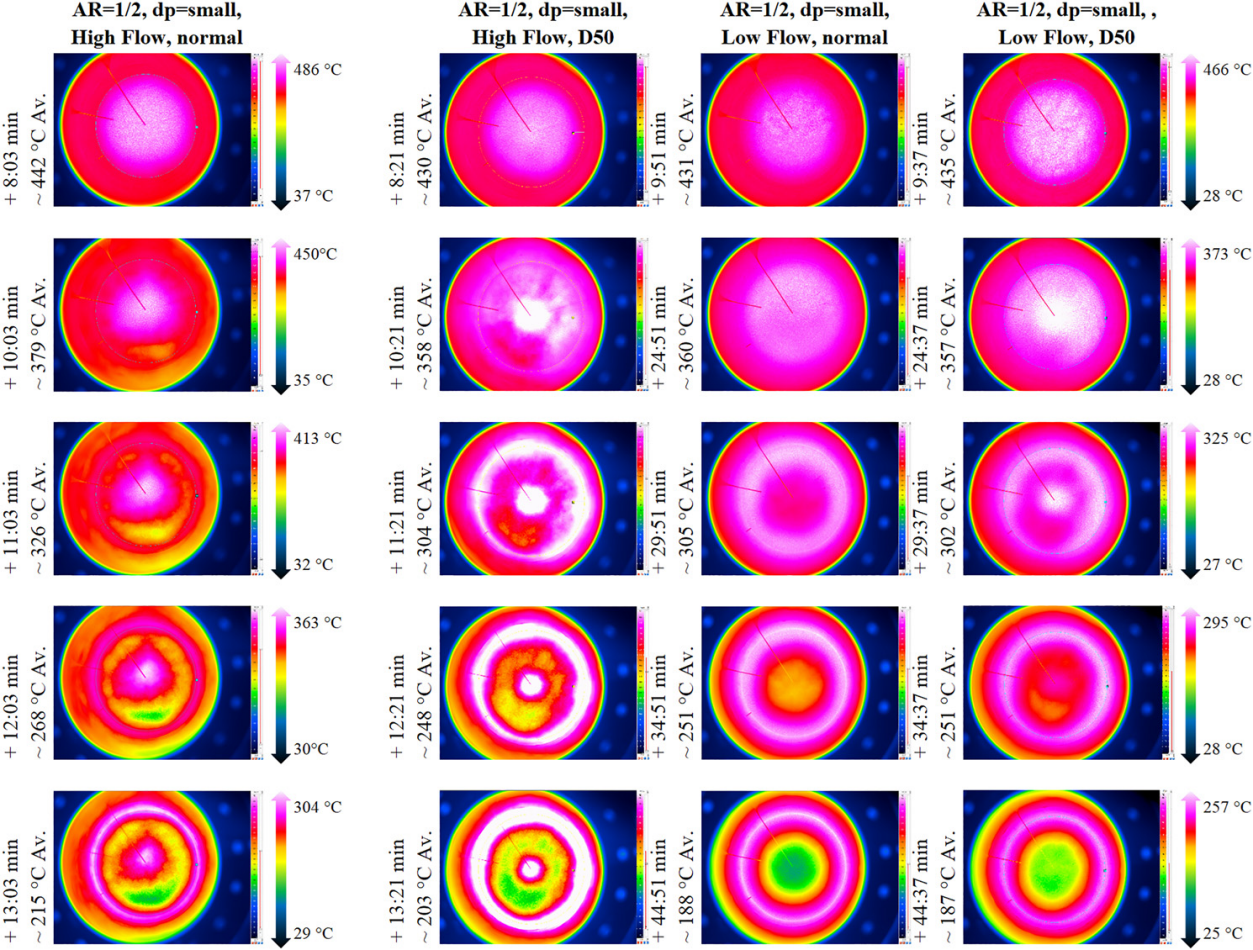
IR snapshots of normal or D50 plate inflow for low and high flow cases (tests from 14^th^ and 20^th^ November, and 5^th^ December).

Complementary exemplary snapshots of IR measurements are shown in Fig. 20 in column one (homogeneous inflow at high flow rate), two (50mm plate inflow at high flow rate), three (homogeneous inflow at low flow rate) and four (50mm plate inflow at low flow rate).

**Fig. 21:**
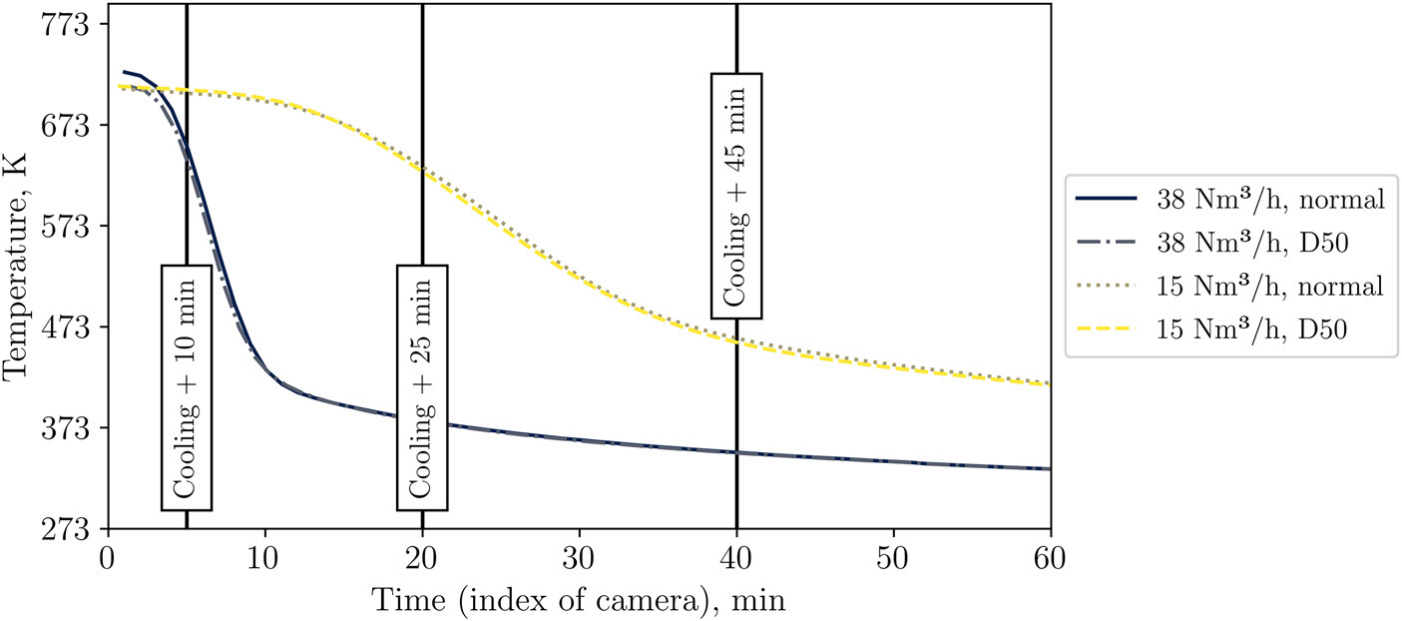
Average packed bed surface temperature vs time for different inflow configurations and flow rates (tests from 14^th^, 20^th^, and 21^st^ November, and 5^th^ December.

To evaluate whether the visible temperature difference on the packed bed surface in Fig. 20 are substantial, Fig. 21 compares the average temperature measured by the IR camera for different flow rates. While there is almost no difference for the low flow case, a slight difference is visible for the high flow one.

### 3.5 Report on ambient conditions

For completeness, we report on ambient conditions, which changed slightly between different experiments and within a single experiment as summarized in Table 4.

**Table 4:** Change of ambient conditions during single experiments.

Test No.	Temperature,°C	Humidity, %	Dew Point,°C	Pressure, hPa
1	15.86 – 21.81	49.23 – 67.36	9.80 – 11.34	1023.3 – 1026.2
2	19.30 – 20.68	56.17 – 64.17	11.36 – 13.03	1018.1 – 1019.0
3	19.54 – 21.73	48.00 – 62.08	9.82 – 12.57	1016.5 – 1019.0
4	15.62 – 20.04	52.84 – 64.94	8.97 – 10.18	1017.5 – 1018.4
5	15.49 – 19.16	38.30 – 51.90	4.07 – 6.67	992.1 – 993.7
6	16.57 – 20.53	39.21 – 46.37	4.84 – 6.26	1003.2 – 1006.2
7	11.69 – 20.17	25.00 – 31.98	N/A (0 – 655.35)	1023.6 – 1026.0
8	15.17 – 20.80	26.19 – 32.61	N/A (0 – 655.35)	1022.9 – 1024.1
9	16.55 – 20.94	29.62 – 41.07	1.67 – 5.55	988.4 – 992.8

## 4 Experimental Design, Materials and Methods

In this section, the experimental design and procedure is presented in detail.

First, the test rig is described in the subsection “Description of Test Stand”. Herein, detailed information about installed devices such as air heater and blower are given. Further, the structure of the experimental setup is outlined, e.g. by giving information about flanges, insulation, and diffusor.

Second, the measurement system is described. Measurement devices are named and their location in the test rig is reported. The accuracy of the measurement devices is presented in Table 5.

**Table 5:** Experimental devices with their accuracy and ranges according to the manufacturer. Rd indicates the reading, FS the full scale and |T| the temperature in Celsius. Thermocouples of type N have been calibrated (c) in-house in 50K steps (increasing) including the whole measurement chain from TC tip to data collection using LabVIEW. Alike, the TC used during the emissivity measurement has been calibrated (c) at 424K,569K and 749K. The TC calibration device itself has been calibrated at the setpoints (SP) 423.15K,573.15K and 873.15K.

Experimental device	Range or Value	Accuracy
Differential pressure	(0−5000)Pa	±1%Rd
Thermocouple (type N, c)	(323−523)K	±4.57K
Thermocouple (type N, c)	(573−823)K	±2.28K
Heater control TC (type K)	(233−606)K	±2.5K
Heater control TC (type K)	(606−1373)K	±0.0075·|T|
Emissivity meas. TC (type K, c)	(423−750)K	±0.5K
Mass flow	$\left(0.3-148\right)\;m^{3}h^{-1}$	±0.015Rd±0.003FS
Ambient sensor: Temperature	(233−398)K	±0.1K
Ambient sensor: Humidity	(0−100)%	±1%Rd
Ambient sensor: Pressure	(300−1100)hPa	±0.1hPa
TC Calibration device, SP No. 1	423.15K	−0.49K(±1.7K)
TC Calibration device, SP No. 2	573.15K	−0.41K(±2.2K)
TC Calibration device, SP No. 3	873.15K	−0.23K(±3.2K)

Third, in the subsection “Metal Screens”, information about the wire meshes, which are used for flow homogenization, is presented.

In the subsection “Particle Properties”, the particulate material is described in its size, material composition, specific heat capacity and thermal conductivity, density and bulk porosity, and emissivity. As the emissivity is evaluated in-house with a test setup adaptable to various materials, the measurement procedure is explained along to the results.

The subsection “Flow Pretests” presents the flow preconditioning due to the previously described metal screens in two steps: a, the measurement method of the fluid flow is explained and b, the resulting flow profile is described.

Finally, the experimental procedure is presented. The paragraph is extended with information on the uncertainty of positioning thermocouples in the bed, as the packed bed has been reinstalled for the determination of measurement insecurity.

### 4.1 Description of test stand

**Fig. 22:**
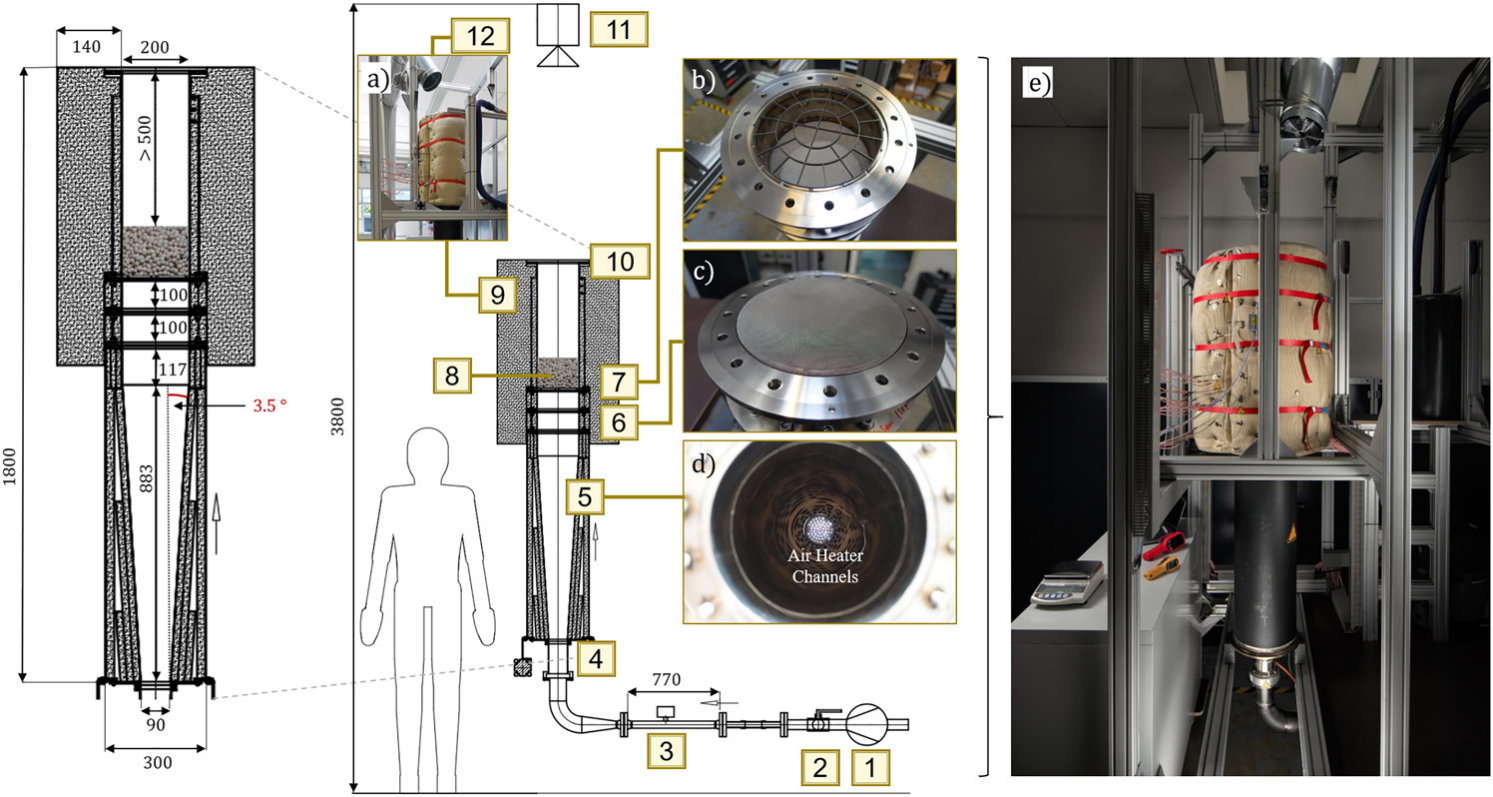
Technical drawing of test stand highlighting the core components with dimensions in mm and set up (2e).

**Fig. 23:**
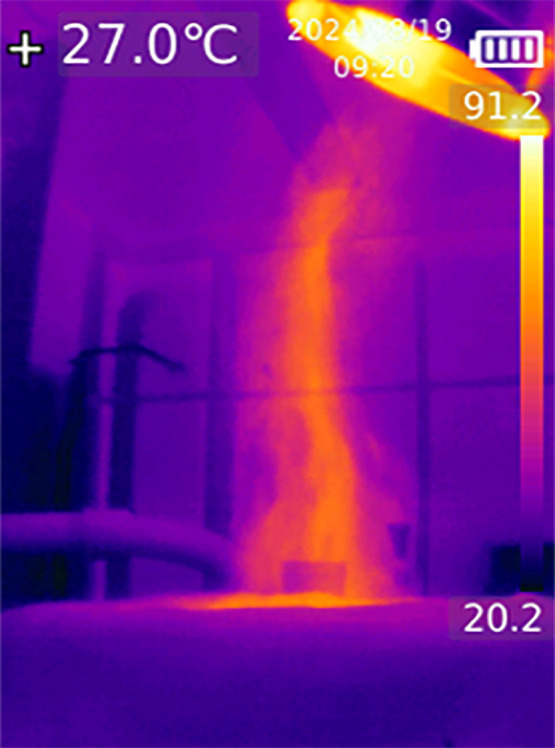
IR image showing the hot fluid outflow above the blind flange (9) near the suction pipe.

A technical drawing of the test rig is shown in Fig. 22. The annotations in the figure indicate different system components, i.e. the air blower (1), a ball valve (2) for pressure tightness tests, a mass flow measurement sensor (3), an air heater (4) that functions additionally as flow straightener, see Fig. 22d, a diffusor (5, Fig. 22d), two metal screens in form of a stainless steel wire mesh (6, Fig. 21c), a particle support structure (7, Fig. 22b) on which an extra metal wire mesh is installed to prevent particles from falling into the air heater, the particle bed (8), insulation (9, Fig. 22a, consisting of two parts which are held together by straps), an optionally installed blind flange (10) used for pressure tightness tests, and the IR camera used for bed surface temperature measurements (11). Furthermore, the suction system (12) for hot air removal is visible in Fig 22a. The height of the suction pipe is carefully selected so that the fluid outflow is minimally affected, see Fig. 23. Moreover, to ensure undisturbed outflow conditions from the packed bed surface, the length of the pipe between packed bed surface and uppermost flange is at least 0.5m in all experiments.

The blower DSC10 by Dassler GmbH^1^ with a maximum rated power of 550W can either operate up to 185mbar or up to $110\;m^{3}h^{-1}$. The inflow length of the mass flow measurement system is 0.57m and outflow length is 0.2m. The 7600W heater PH92 by Hertz GmbH^2^ can operate up to a maximum temperature of 923K. Its outlet with a diameter of 90mm is attached to the diffusor flange.

The 883 mm long diffusor consists of a 0.2mm thick stainless steel (1.4301) sheet, formed into a capped conical shape with $7{\;}^{\circ }$ opening angle and 198mm upper inner diameter, has a floating bearing, and sits in an outer vessel of inner diameter 300 mm. The space between diffusor and outer vessel is almost completely filled with a glass fleece^3^ (binder-free, mechanically bonded needled nonwoven made of E-glass according to DIN 1259) with a thermal conductivity of $0.037\;Wm^{-1}K^{-1}$ at 323K and $0.102\;Wm^{-1}K^{-1}$ at 773K that can be operated up to 873K. Each installed mat has a thickness between 20 and 25 mm with a density of between 150 and $180\;\text{kg}\;m^{-1}$. Data about the specific heat capacity is not available.

The diffusor leads to a flange (stainless steel 1.4301, mass of 3kg, used 2 more times within the system, see Fig. 22) in a pipe system. Stainless steel pipes are 2mm thick and have an inner diameter of 200mm. The distance between beginning of diffusor and first flange is 1000mm.

Each flange fits a metal screen that is used to homogenize the fluid inflow, see the following subsection. The distance between the screens is 0.1m. The pipe system is, first, insulated with 0.02m thick glass fleece mats, and, second, with a 0.14m thick insulation mattress by the company Weihe filled with a stone wool mat PAROC Pro Mat 100. This material has a thermal conductivity of $0.039\;Wm^{-1}K^{-1}$ at 323K, $0.059\;Wm^{-1}K^{-1}$ at 473K, and $0.131\;Wm^{-1}K^{-1}$ at 773K. Information about density and specific heat capacity is not available.

**Fig. 24:**
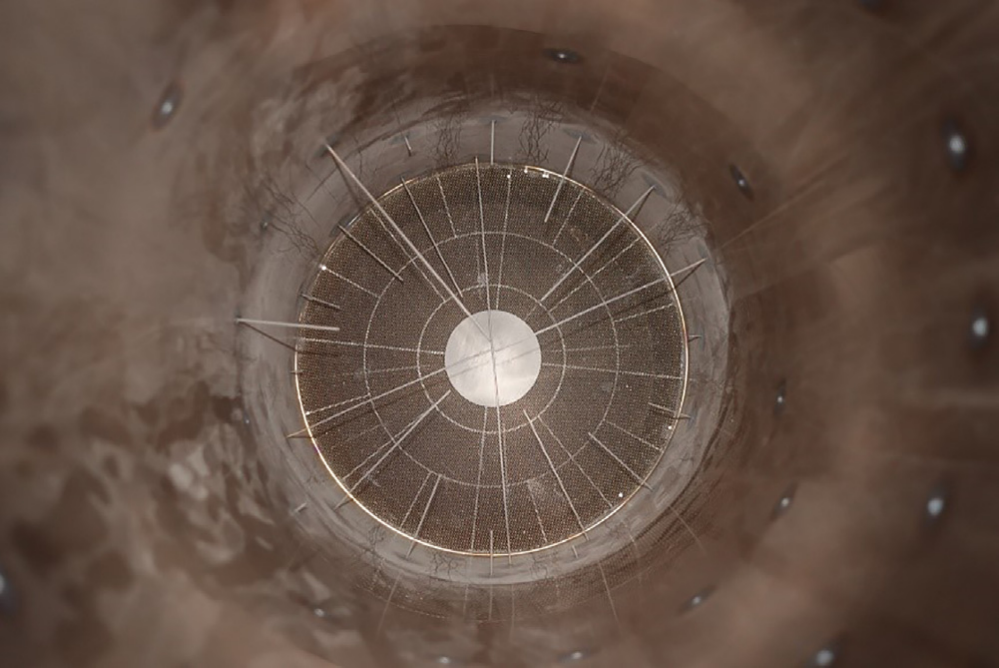
Particle support structure with D50 plate on top.

The particles are placed on a screen which is supported by a particle support structure (see Fig. 24 and left-hand side of Fig. 26) of material type 1.4762 with a mass of 0.105kg. On top of this particle support structure, a 1mm thin steel (1.0038) plate with a diameter of 50mm can optionally be installed, see Fig. 24. This plate functions as a blind spot so that the fluid does not enter homogeneously into the packed bed. In this way, data for a validation case with inhomogeneous flow can be generated.

### 4.2 Measurement system

An overview of measurement devices can be seen in Table 5.

The calibration device of the manufacturer “ISOTECH und Klasmeier” is of type “Pegasus Site” and equipped with a type R TC. Calibration of the calibration device has been done in an external company at temperature setpoints (SP) of 423.15, 573,15, 873.15 K according to policy “DKD-R 5-4:2018” and “KA16-10-01:2018-01”. In Table 5, the difference between calibration device and calibration temperature and its measurement accuracy according to policy “EA-4/02 M:2022” (see brackets) is shown.

**Fig. 25:**
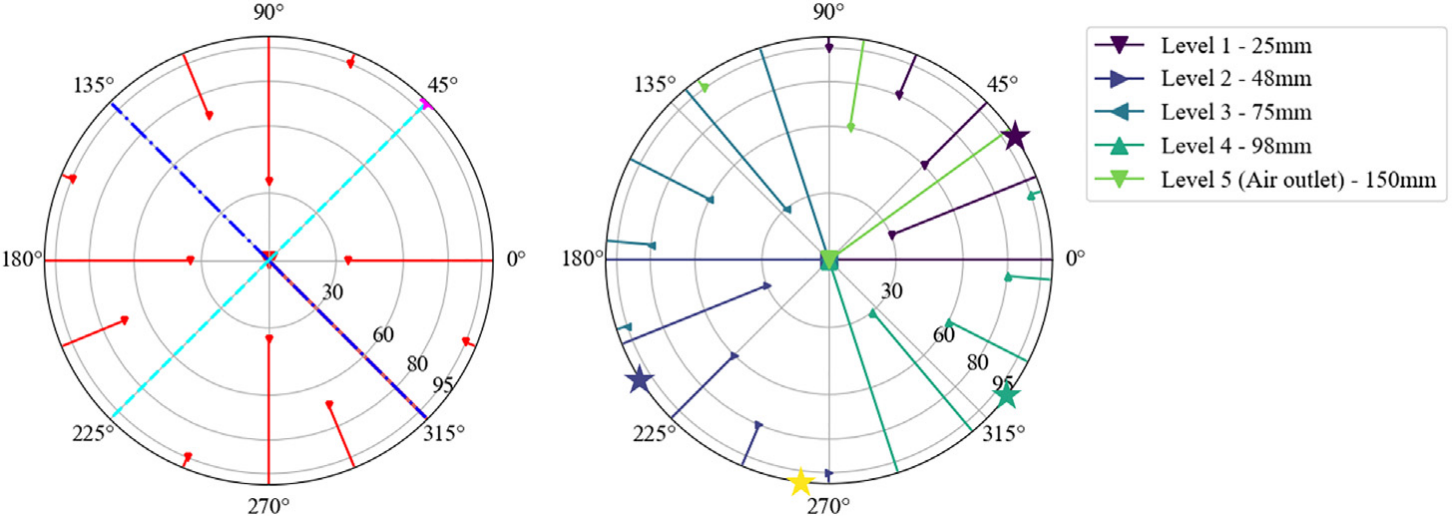
Left polar plot: Location of TCs 15mm below bed (red), of axis of velocity measurement (cyan, blue) and of differential pressure measurement below bed (pink star at 45°). Right polar plot: Location of TCs within bed and circumferential position of insulation TCs (stars).

**Fig. 26:**
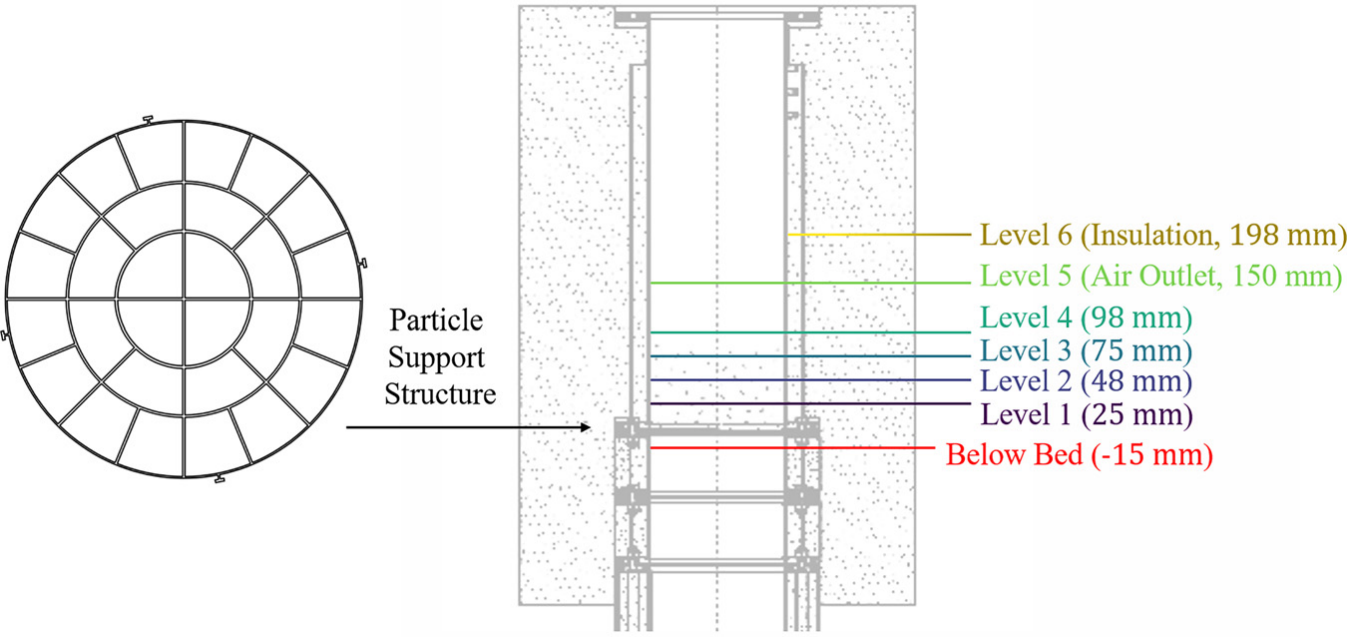
Visualization of vertical placement of TC in packed bed and in insulation.

To evaluate different bed configurations, several type N TC (class 2) with 0.001m wire diameter and 500mm TC length are installed below, within and above the packed bed, as seen in Fig. 25 (top view) and Fig. 26 (vertical view).

Four TC measure the steel temperature of the pipe outside on level 1, 2, 4 and 6 (198mm). Their circular position is each between the thermocouple feedthroughs, from where thermocouples are normal to the pipe inserted into the packed bed. In Fig. 25, the steel thermocouple position is marked with a star (yellow: level 6) on the right polar plot of the figure.

Note that the steel temperature is measured by inserting the TC between the steel pipe outside and a tight fitting aluminium foil, which was installed to avoid the formation of chromate. The insulation temperature is measured at 100mm insertion depth (measured from the outside of the insulation) on level 1, 2, 4, and 6 with one TC on each level, and additionally at 25mm insertion depth on level 2.

Above the heater, 1216mm below the packed bed, a type K TC is installed. This TC is used in the PID control of the outlet temperature of the heater.

Ambient conditions (temperature, humidity, barometric pressure) are monitored with the sensor “S-THP-01A” by Antratek^4^ nearby.

The pressure drop in the packed bed is measured with a differential pressure sensor “IDS2” from ICS Schneider Messtechnik GmbH^5^. Functionality of this device has been tested in cold conditions, and the measured pressure drop of different packed bed heights complies well to the Ergun equation so that they are not reported here. However, during hot tests, the pressure drop is measured and to be found in the raw data. Mass flow in the system was measured by the “IVA 520 thermal mass flow sensor” by company ICS Schneider Messtechnik GmbH.

The gas velocity was measured during the cold tests only because the measurement method used is based on thermal measurement principles. Flow measurement methods such as Laser Doppler Anemometry, pressure sensitive paint, pitot probes, hot wire anemometry, particle image velocimetry or other coating and tracer solutions would have been either too complex to install given the limited additional information that they introduce or are in general not applicable. Methods such as velocity measurement after flowing through the packed bed have not been applied due to measurement uncertainties as the measured velocity is highly dependent on the distance between flow outlet, i.e. bed surface, and probe. Although some authors mention flow splitters as solution to this common problem, their application is also limited [10].

Velocity measurement took place without installed packed bed and the pipe system around the packed bed due to accessibility reasons. Further information about the measurement is described in the subsection “Flow Pretests”.

A long range (7.5−14)μm IR camera "VarioCAM HD head 800 research" from InfraTec^6^ is used. The detector is a non-cooled focal plane array detector with a resolution of 1024 × 768 pixels. The temperature measurements (up to 1473K) have a resolution (noise equivalent temperature difference) of less than 0.02K at 303K with an uncertainty of ±1K or ±1% (whichever is greater). The installed lens is a 60mm tele lens with an angle resolution of 0.28mrad and a minimum focal length of 1m. The focus is set manually prior to each measurement, and the temperature range, for which the camera was calibrated, is chosen automatically by the measurement software. The camera was set to take 1picture per minute, whereas this has been changed to 2pictures per minute in some cases with higher flow volume or to 30Hz in case of emissivity measurements. The distance of the camera to the outflow flange is 0.87m. Distance between outflow flange and packing is >0.50m.

### 4.3 Metal screens

All installed metal screens (high temperature stainless steel 1.4841) have a wire diameter of $d_{w}=0.4\;\text{mm}$ and a mesh size of $w_{m}=0.63\;\text{mm}$ with a screen porosity of $\beta_{s}=0.133$. In total, three screens are present in the system, see Fig. 2. The two lower screens aim at flattening the velocity profile and the third screen is used as particle support. Regarding the flattening screens, a typical $\beta_{s}$ for screens in wind tunnels with the purpose of turbulence reduction is between 0.5 and 0.8 [11]. According to Mehta et al. [12], too low porosity might result in spatial and temporal flow instability. Nonetheless, the very low porosity screens were chosen due to a) the available material suitable for high temperature applications, and as b) the pressure drop due to the screens is not a limiting factor in the presented system as it might be in a wind tunnel. Additionally, the presented screens were able to flatten the velocity profile sufficiently as shown in a later section.

**Fig. 27:**
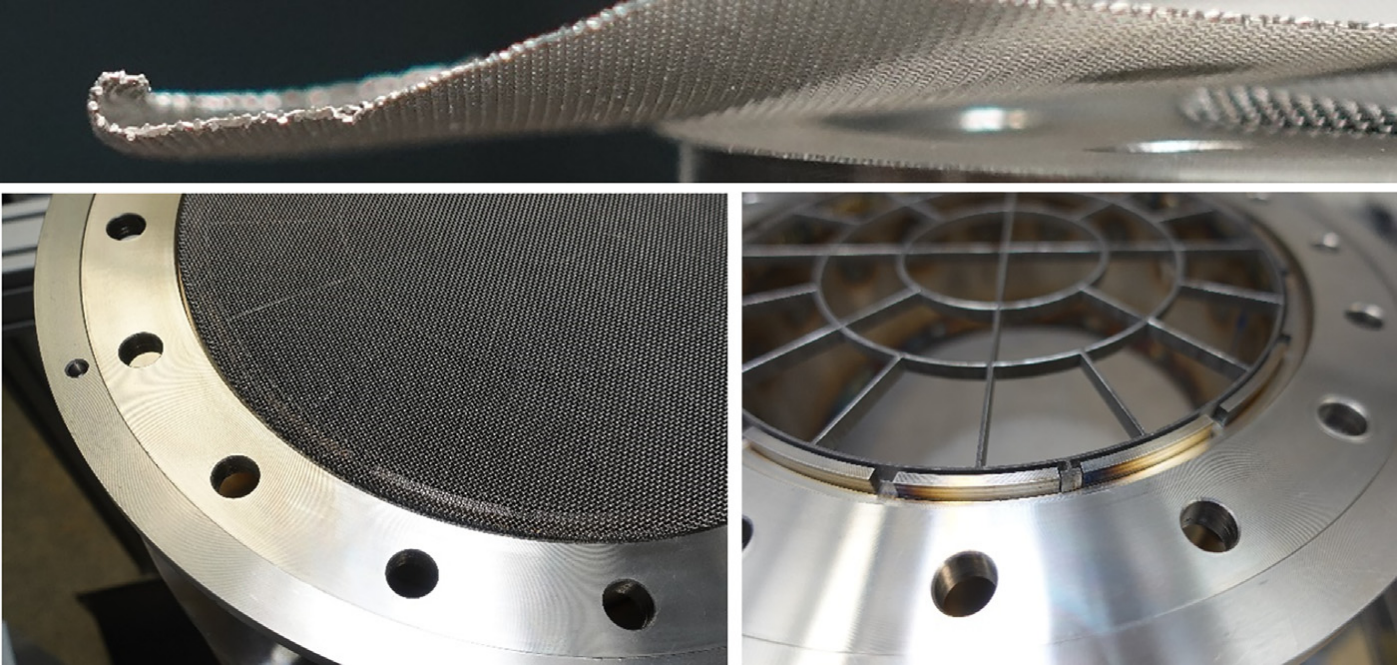
Close-up on rolled edge of screen (top), screen in groove (bottom left), groove in flange (bottom right).

All screens have a rolled-up edge, which is fit into a groove of the flanges (see Fig. 27). By this measure, any elongation and therefore irregularity due to heating of the screens is supposed to be reduced in its effect. At this point it should be mentioned that the screens themselves are not perfectly in plane due to the manual manufacturing process.

To avoid bypassing effects as explained in subsection “Flow Pretests”, high temperature silicone is used in sealing of eventual gaps to avoid velocity peaks close to the wall.

In order to use the experimental data of the main experiment for validation purposes, various boundary conditions have to be known such as information about particle properties and flow profiles, which will be presented in the following subsections.

### 4.4 Particle properties

#### 4.4.1 Size

**Fig. 28:**
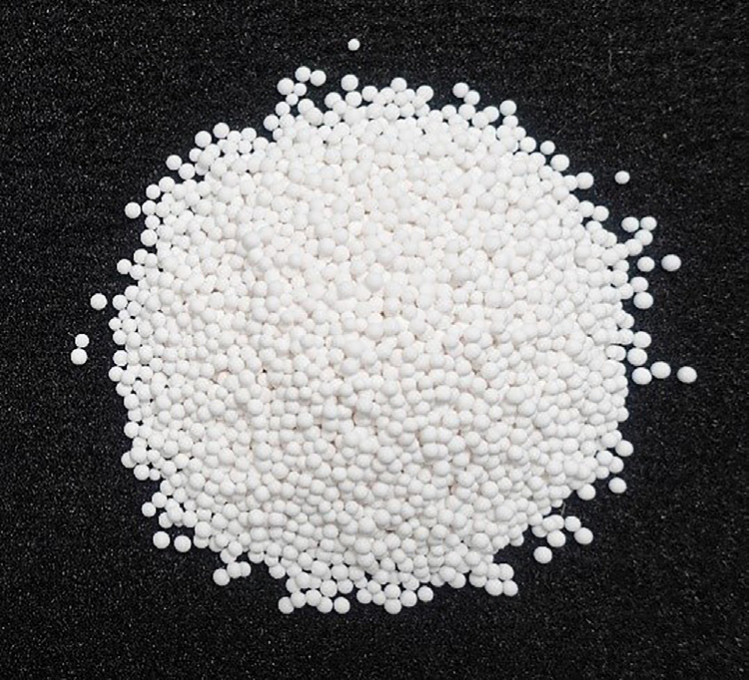
Particles used in experiments.

The particle bed is formed by particles of the company Mühlmeier GmbH & Co. KG^7^ with a Sauter diameter $d_{s}=2.13\;\text{mm}$, see Fig. 28.

**Fig. 29:**
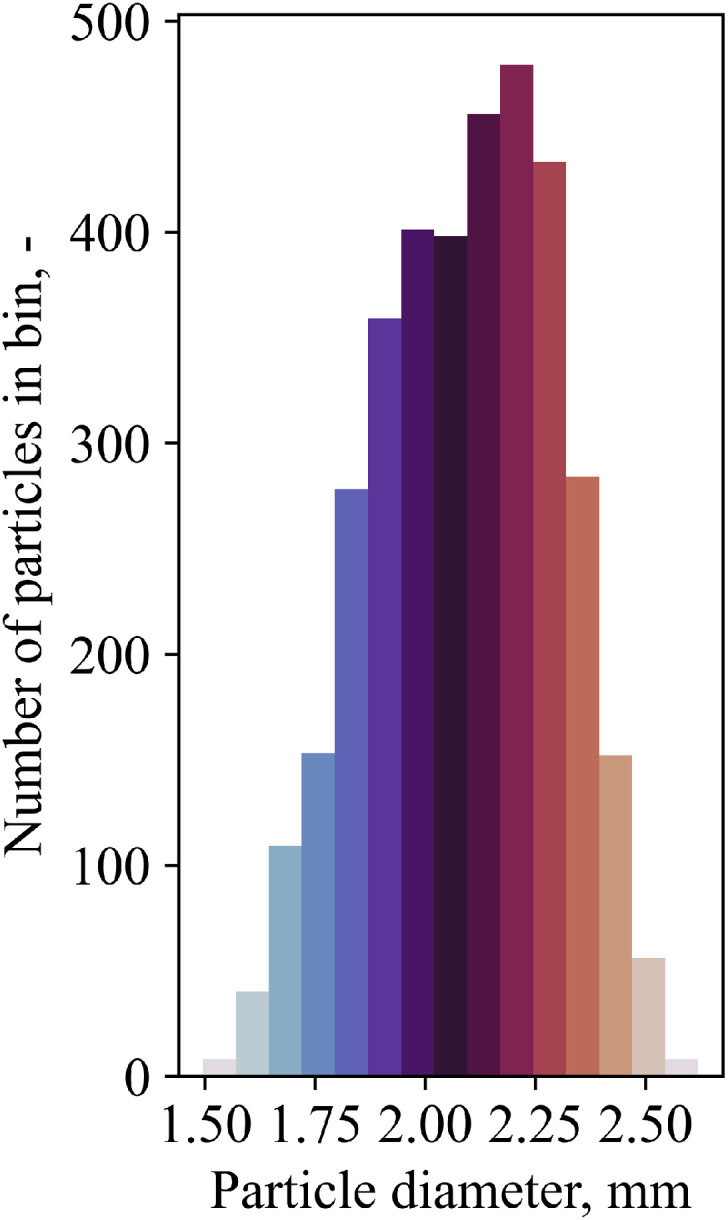
Size distribution of particles.

The particle diameter together with other properties, see Table 6, was evaluated with a random selection of 3614particles. The particles were placed on a rough surface next to a scale with an uncertainty of 0.1mm. The rough surface is to reduce artificial effects by rolling to a more stable position. Pictures of a digital camera with a focal length of 70mm were processed in the OpenSource software ImageJ^8^, i.e. setting scale, converting to 8bit figure, setting threshold to differentiate between particles and background, watershed filter to separate touching particles, cropping and analyzing. The resulting histogram, which can for instance be used in DEM simulations, is shown in Fig. 29, and the raw data is presented in folder 0 of the dataset.

**Table 6:** Results of particle size analysis using the software ImageJ.

Parameter	Value
Sample Size N	3614
Arithmetic mean diameter ${\bar{d}}_{\text{mean}}$ in mm	2.091 ± 0.003
Minimal area-based diameter $d_{A,\;\text{min}}$ in mm	1.50
Maximal area-based diameter $d_{A,\;\text{max}}$ in mm	2.62
Sauter diameter $d_{s}$ in mm	2.13
Mean circularity ${\bar{S}}_{\text{mean}}$	0.959 ±0.000

#### 4.4.2 Material composition

The particle properties were obtained with an energy dispersive X-ray spectroscopy (EDS) in-house. They consist of $90\%\;Al_{2}O_{3},$ $4.6\%\;\text{Si}O_{2},\;3.1\%\;\text{CaO}$ and 2.0%MgO. The content of $K_{2}O$ is less than 1%, and, hence, is neglected in further analysis given the limited amount.

#### 4.4.3 Specific heat capacity and thermal conductivity

Thermal properties of the particles were calculated using data sources in [13] and [14] together with the composition, respectively. Fitting leads to Eq. (1) of the thermal conductivity $\lambda_{p}$ in $\text{Wc}m^{-1}K^{-1}$ and to Eq. (2) of the specific heat capacity $c_{p,p}$ in $J\;kg^{-1}K^{-1}$ for a data range between (283−823)K with the temperature T in kelvin.

(1) $$\lambda_{p}=3.6109\cdot 10^{-12}\cdot T^{4}-9.7658\cdot 10^{-9}\cdot T^{3}+1.0228\cdot 10^{-5}\cdot T^{2}-5.1344\;\cdot 10^{-3}\cdot T\;+\;1.1864$$

(2) $$c_{p,p}=-8.1716\cdot 10^{-9}\cdot T^{4}\;+\;2.1620\;\cdot 10^{-5}\cdot T^{3}-\;2.1801\cdot 10^{-2}\cdot T^{2}+\;10.3580\cdot T\;-\;8.8547\;\cdot 10^{2}$$

#### 4.4.4 Density and bulk porosity

Density of single particles was measured based on the Archimedes principle with 10 samples, leading to ${\bar{\varrho }}_{p}=\left(3.650\pm 0.015\right)\;g\;cm^{-3}$.

The bulk porosity was measured by pouring a measured mass of particles in a defined cylindrical volume ($800\;cm^{3})$ with 100mm diameter. The measurement was repeated 10 times, resulting in ${\bar{\phi }}_{\text{bulk}}=0.377\pm \;0.003$ and ${\bar{\varrho }}_{\text{bulk}}=\left(2.273\pm 0.010\right)\text{gc}m^{-3}$.

#### 4.4.5 Emissivity

Knowledge on the emissivity ε is necessary as already an error of a few percent in the knowledge of ε leads to large error in the infrared temperature reading [15].

**Fig. 30:**
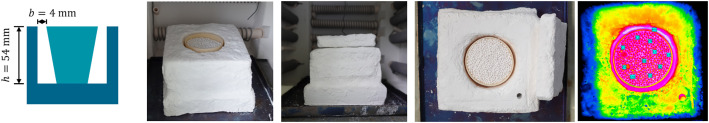
Setup of emissivity measurement. From left to right: a) Crucible dimensions, b) cuboid insulation box in chamber furnace, c) insulation box with lid, d) top view on crucible in insulation box, e) example figure taken by IR camera.

To measure the emissivity of the particles at elevated temperatures, various techniques and measurement devices exist. A summary is presented in [15]. Within this pre-study, a comparison method is used. Precisely speaking, a chamber furnace and an IR camera (which is the same as in later measurements of the packed bed surface) are utilized. Particles were poured in a ceramic crucible which was then placed into a cuboid box of insulation material. The crucible does not fit perfectly, and thus leaves a small gap around the crucible, see Fig. 30 a and d. With a gap width of $b_{\text{gap}}=4\;\text{mm}$ and depth of $h_{\text{gap}}=54\;\text{mm}$, this gap is used as black body reference as $h_{\text{gap}}\;\gg \;b_{\text{gap}}$. The emissivity of the black body reference $\varepsilon_{\text{gap}}$ is calculated based on view factors with a normal total emittance of the black body material of $\varepsilon_{\text{bb}}=0.7$ [14]. This leads to an emissivity of $\varepsilon_{\text{gap}}\approx 0.99$. Further, to avoid heat loss during the short transport from the furnace to the nearby measurement location with IR camera, the box is equipped with a removable lid of insulation material.

For each measurement, the cuboid with the open lid was placed into the chamber furnace. A type N thermocouple (TC) is then placed into the particles. The furnace is closed and the particles are heated until a steady state temperature is reached. Afterwards, the furnace is opened and the lid with a small hole as TC feedthrough is placed on top. Heating is continued until, again, a steady state temperature is reached. Then, the still closed insulating box is removed from the furnace and put in a designated place, where the IR camera described above with a fixed focus already records the surface temperature. The effective wavelength of the camera can be approximated to $\lambda_{\text{eff}}=10.3\;\mu m$ for an object temperature of 723K according to [15]. Eventually, the lid is removed and the first image recorded by the camera (30 Hz) is used for analysis. Using the first available image reduces temperature errors due to cooling during transport from furnace to measurement location or due to convection losses. Note that the heating of the sample takes several hours, and the relocation of the insulation box, removal of the lid and the IR measurement happens in less than a minute.

For analyzing the image, either single particles (between 7 and 13 per evaluation) or the whole packed bed surface is selected. Parts around the crucible, where the gap is present, appeared to have the highest temperature in the IR image and are thus selected as reference. Now, by reducing the emissivity value of these selected particle areas until the temperature matches the reference temperature, the emissivity is evaluated. Several temperatures (394K, 423K,569K, 746,747 and 749K) were measured with the measurement at 569K being repeated two times.

**Fig. 31:**
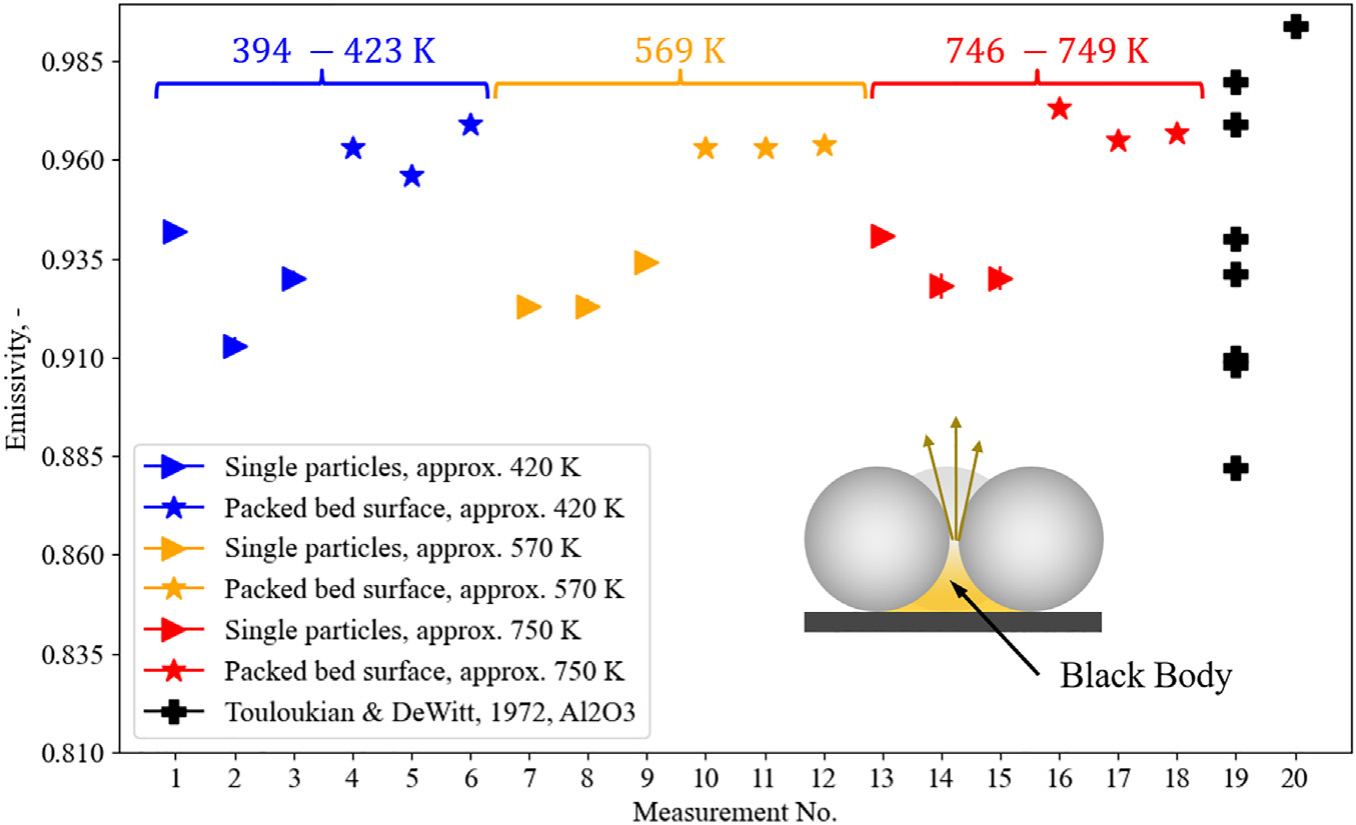
Results of emissivity measurement.

The results are shown in Fig. 31. The indicated errors are statistical errors, in other words not the error due to possible uneven temperatures within the probe or the reference area. A substantial difference between single particles and the bed cross section is visible, resulting from the effect of small black body cavities between single particles as illustrated in the same figure.

The measured values are found within the range of literature values: No. 19 in Fig. 31 describes different samples of $Al_{2}O_{3}$ presented in [14] for a temperature range of (813−885)K and a wave length of (9.97−11)μm. The single value depicted in No. 20 is reported for a temperature of 293K and a wave length of 10.3μm. Values of $\text{Si}O_{2},\text{CaO}$ and MgO are in the range of 0.85−0.94. As their fraction is rather low, they are not shown in Fig. 31.

Not shown in the figure is an additional measurement conducted with a camera with a wavelength of (2−5)μm. As the material properties depend on the measuring wavelength, the measured value is significantly lower, namely 0.434. Again, this agrees to literature values [14].

Besides statistical errors, errors due to inhomogeneous temperature of the probe can be substantial. Regarding the calculation of these errors, readers are referred to Bernhard et al. [15].

### 4.5 Flow pretests

#### 4.5.1 Flow pretest description

To test pressure tightness in the system, the whole system was heated once to the maximum operating temperature until it reached steady state. The test rig was then cooled down again. Then, a blind flange and a sealing were installed at the top of the test rig. The blower was then operated at full throttle, the ball valve was closed and the pressure drop in the system was recorded. From the drop of pressure over a certain time, a leakage rate can be calculated. Relating this leakage rate to a volumetric flow of $15\;Nm^{3}\;h^{-1}$, a leakage rate of less than 2% is present.

**Fig. 32:**
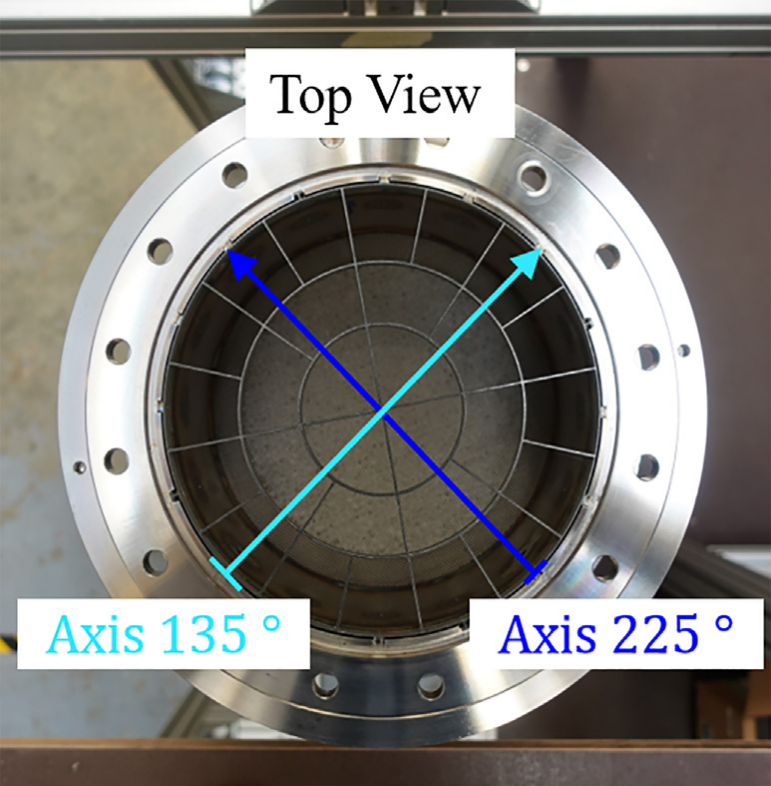
Axes used for velocity measurement. The flow is coming from the right.

After evaluation of the pressure tightness, the metal mesh screens performance has been evaluated by measuring the fluid velocity using the “TSI8455” velocity transmitter by Driesen+Kern^9^ in a range of $\left(0.125-1.000\right)\;ms^{-}1$ with an accuracy of ±2% of reading ±0.5% of measurement range and with a time constant of 10s. The velocity was measured shortly below the particle bed holding flange along two different axes, as can be seen from Fig. 32. From the starting point (lower left and lower right) the probe is inserted in small steps (10mm) into the pipe. Due to a minimal insertion length, the first measurement takes place at 19mm until, eventually, the probe head touches the wall. Here, the measurement location itself is at 189mm.

During the measurements, the packed bed and the respective pipe was not installed due to accessibility reasons. The measurements were performed in a cold state only due to the limitation of the measurement device, which uses a thermal measurement method.

#### 4.5.2 Flow pretest results

**Fig. 33:**
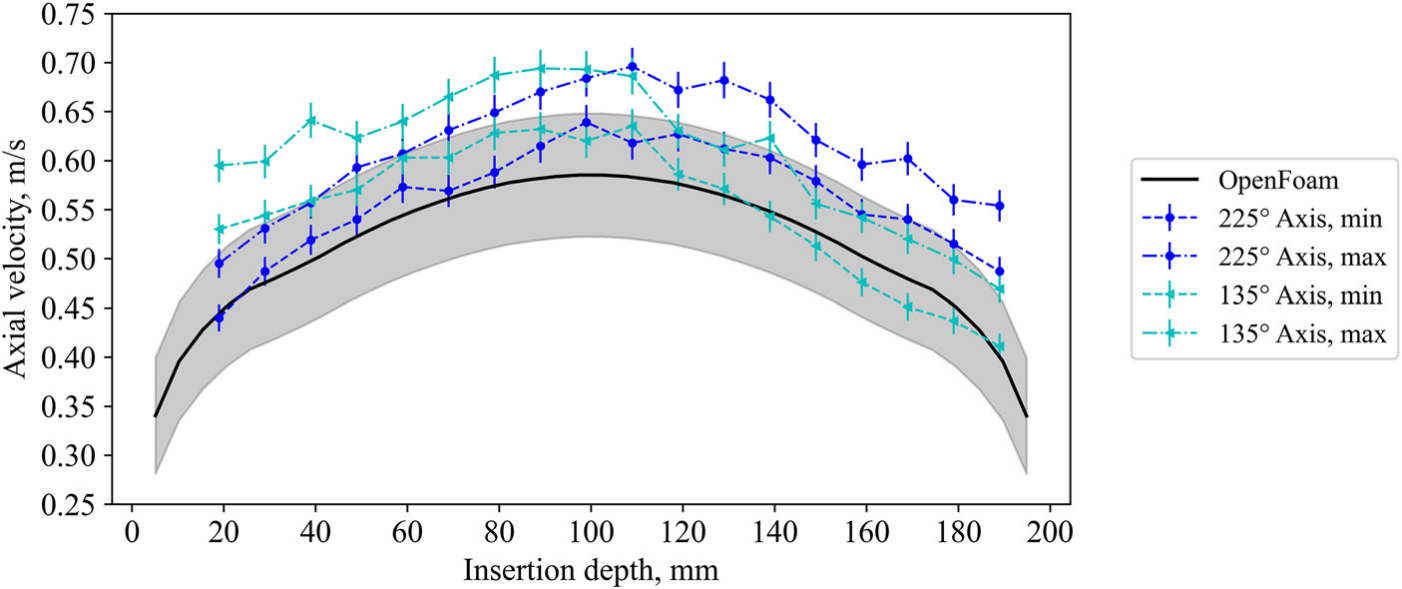
Velocity measurement without screens installed for a volumetric flow rate of approximately $50\;{\mathrm{Nm}}^{3}h^{-1}$. The measurement starts at an insertion depth of 19mm, which is the minimal insertion length of the probe to allow measurements. Note that the walls are at 0mm and 200mm.

First, the velocity was measured without any screens installed, see Fig. 33. Here, the grey error region around the OpenFOAM data is calculated on the mass flow sensor accuracy as indicated in Table 5. Vertical error bars around measurement points are calculated on the velocity sensor accuracy as reported in the previous subsection. Calculations are performed in Python, see the file “plot_velocity_flow-pretest.py”. Note that no data was normalized or filtered in the whole experimental campaign, unless stated otherwise.

The general profile complies with the typical empty pipe profile. Moreover, it can be seen that for each axis a minimum and maximum value is indicated. This is due to the strong fluctuation of the measurement signal at each position.

**Fig. 34:**
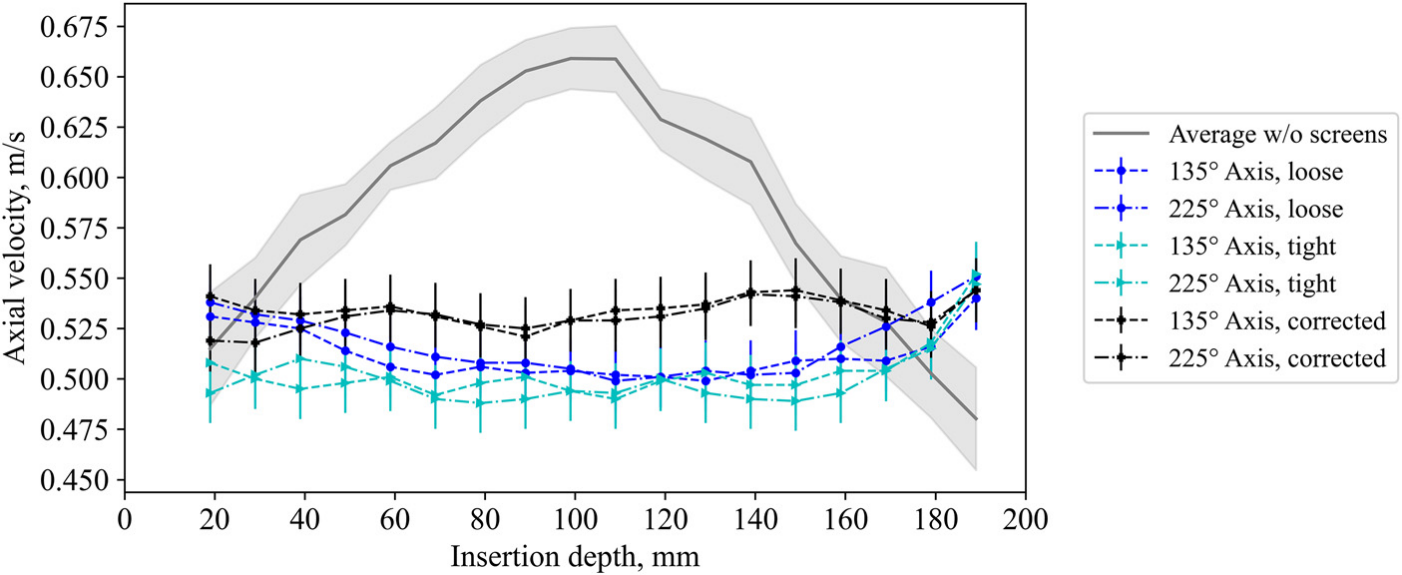
Velocity measurement with screens installed for a volumetric flow rate of $50\;{\mathrm{Nm}}^{3}h^{-1}$. The walls are at 0mm and 200mm.

Furthermore, the velocity was measured with installed screens, see Fig. 34. Three tests (loose screens, sealed screens, sealed screens with probe correction) have been performed. For each axis and position, no temporal variation in the reading value could be detected.

**Fig. 35:**
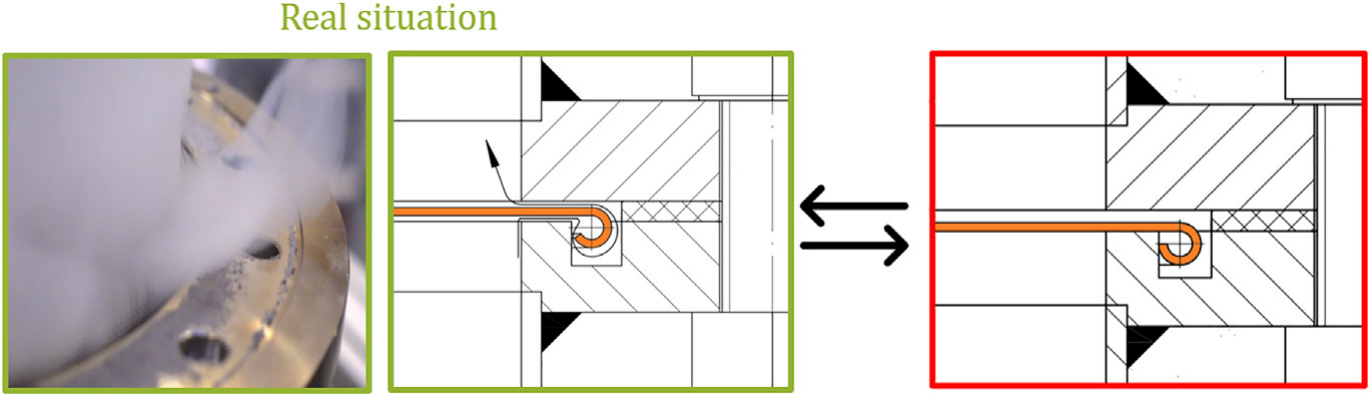
Image of bypass flow visualized with a fog machine and a drawing with the explanation for it.

With loose screens, the velocity close to the walls (located at 0mm and 200mm insertion depth) is higher than the velocity in the center. This is reasoned in the presence of a bypass flow, which was visualized by artificial fog, see Fig. 35. The bypass is due to slightly uneven screens, resulting in the fact that they are not everywhere in contact to the steel, and thus, little gaps are present which lead to a path with less resistance.

**Fig. 36:**
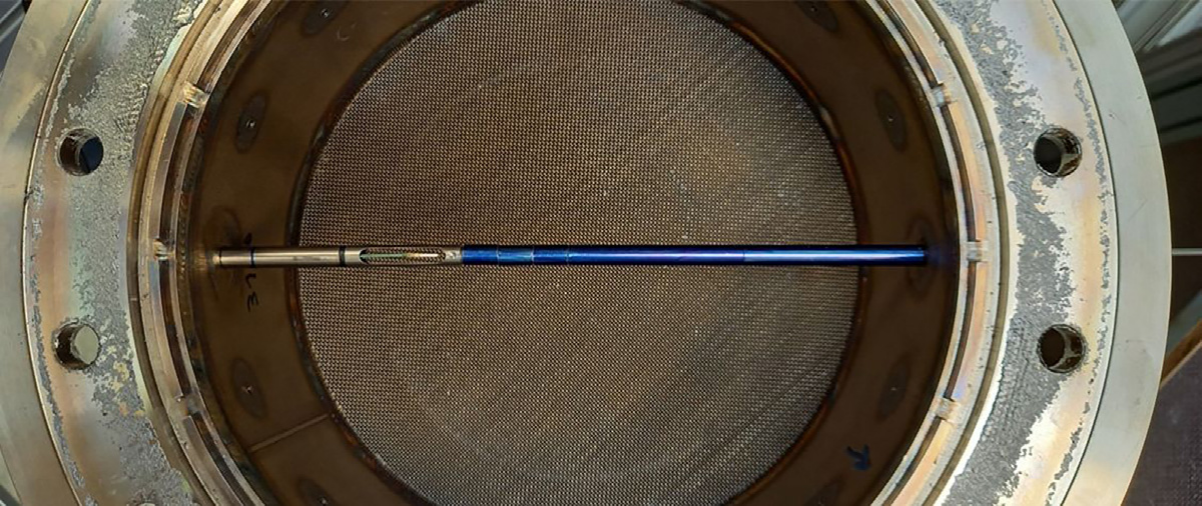
Realization of probe extension using sleeves (blue, each 10 mm) from the opposite end.

By sealing the screens with a high temperature silicone (Ulfalux Thermo-Elasto), the fluid velocity decreases for low insertion depths (compare blue and cyan line in Fig. 34). The peak at the right of Fig. 34 at full insertion length of the probe can be explained by the influence of the probe itself, which changes the free cross section of the pipe during insertion. Hence, a probe extension, see Fig. 36, was used for final measurement, which results in a nearly perfectly flat profile (black line in Fig. 34). Small differences could arise due to the very low porosity of the screen and are within the measurement uncertainty. Note that the profile for lower flow rates is similarly flat and thus not reported here.

Finally, keep in mind that the results apply for a cold measurement. In a hot test, the velocity may be different due to a different temperature profile across the axes.

### 4.6 Experimental procedure and information on thermocouple positioning

Having presented the results of necessary flow pretests, the actual experimental procedure can be explained. Each experimental test starts from ambient or close to ambient (less than 323K within the packed bed) conditions.

A packing height of 0.1m is used throughout all cases, equaling an aspect ratio of 0.5, which translates to (7350±5) g packing mass. An existing packed bed is either used again or, e.g. in case of the reproduction tests, renewed. In the latter case, the TC positions have been checked in between tests using a 3D printed gage, so that circumferential positions are within ±0.0015mm. Note that axial positioning is more difficult, and hence, differences between ±0.005m are possible. This is especially relevant for the TCs just below the bed top surface. During filling of the bed, it is ensured that TCs are covered with particles, and the number of particles on top of the TCs varies between 1 and 5 $d_{p}$.

After preparation of the packed bed, a constant mass flow ($38\;Nm^{3}h^{-1}$) is introduced into the system. After a short stabilization time, the air heater is turned on and its outlet temperature is controlled by a PID controller to the desired value, which is 823K if not stated otherwise. The system is heated until a nearly steady state behavior is reached. In other words, the cooling was initiated once the gradient of the close-to-wall thermocouple in the top surface layer of the packed bed (name in raw data: TR404-108e), which is the most inertial thermocouple, was less than $1\;K\;\text{mi}n^{-1}$ (evaluated within the last 15 minutes). This criterion is later referred to as “end of heating” criterion. Then, the air heater is switched off and the desired mass flow for the cooling phase is set. The packed bed is cooled until TR404-108e reaches temperatures below 373K, and a safe switch off of the system can be guaranteed.

## Limitations

**Fig. 37:**
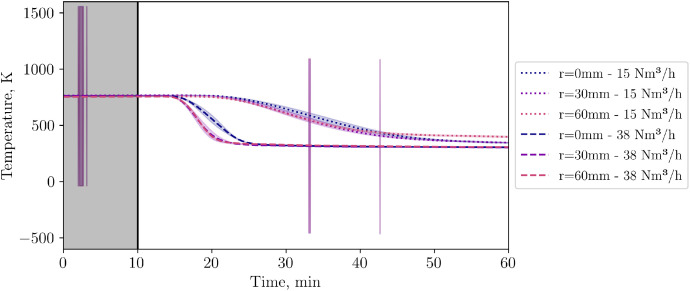
Thermocouples in level 3. In one test, one ADAM module appeared to have failures for a few seconds (temperatures up to 1500 K), which can be clearly identified as (low and high) peaks. After and before the peak, the TC reading shows reasonable values.

Thermocouple data collection was done with Advantech ADAM modules and an improved in-house cold junction compensation compared to the standard one. Unfortunately, one of these modules appeared to have randomly electrical failure as shown in Fig. 37. Outliers are not removed from the raw data, and can clearly be identified. As the data behaves as expected before and after the outliers, no influence on the remaining data quality is expected.

Besides, the authors would like to remind on the following aspects, which could partially influence the results obtained:

• The pressure tightness was checked once in cold state only.
• The fluid velocity close to the fluid entrance into the packed bed was measured in cold experiments only and without a particle bed on top due to limitations in available measurement devices and accessibility of the measurement system. Furthermore, the velocity profile has been presented for a fluid flow rate of $50\;Nm^{3}h^{-1}$.
• Particle emissivity was measured in-house. Here, it was assumed that the heat loss happening between sample removal from the furnace and opening of the sample’s lid / taking of the first picture is negligible. The influence of reference material, i.e. the insulation material around the gap, has not been evaluated as the measured values agree with measurement data and deviations due to this material influence are assumed to be acceptable.
• Naturally, thermocouples within the packed bed measure not only the fluid or the particle temperature but a mixture. Radiation effects due to nearby walls might have a not further assessed impact on the reading value.
• Thermocouple positioning has been checked between experiments with a 3D printed stencil. However, especially the vertical alignment is difficult to control, leading to deviations of up to ±0.005m. Additionally, the number of particles on top of the thermocouples in level 4 varies between 1 and $5\;d_{p}$. Thus, radiation losses from the top layer of the packed bed might influence the measured temperature depending on the amount of particles shielding the thermocouple.
• The air heater has been replaced between experiments (for further explanation see section “Experimental Design, Materials and Methods” of the manuscript).
• Measurement uncertainty for the D50 cases with inhomogeneous inflow is not re-evaluated but taken from the reproducibility experiments with a homogeneous inflow.
• Finally, the authors would like to mention that the insulation temperature was measured at 4 different axial positions. Installation of additional TCs would have improved accuracy of heat loss estimation, but was out of the scope of this work.

## Ethics Statement

The authors have read and followed the ethical requirements for publication in Data in Brief and confirm that the current work does not involve human subjects, animal experiments, or any data collected from social media platforms.

## CRediT Author Statement

**Anika Weber:** Conceptualization, Methodology, Formal analysis, Investigation, Resources, Writing - Original Draft, Writing - Review & Editing, Visualization. **Sebastian Schürg**: Methodology, Investigation, Writing - Review. **Timo Roeder**: Methodology, Writing - Review & Editing. **Johannes Grobbel**: Conceptualization, Methodology, Writing - Review & Editing, Supervision, Project administration. **Martina Neises-von Puttkamer**: Writing - Review, Supervision, Project administration, Funding acquisition. **Christian Sattler:** Writing - Review, Supervision, Project administration, Funding acquisition.

## Data Availability

Mendeley DataExperimental study of heating and cooling of a shallow packed bed suspected to air temperatures between 300 and 773 K (Original data).

## Footnotes

1 https://www.dasslerventilatoren.info/contents/de/d29_Hochdruckgeblaese.html.

2 https://www.herz-gmbh.com/de/produkte/lufterhitzer-geblaese-zuendgeraete/rohrlufterhitzer-160.

3 https://www.steinbach-ag.de/thermische-und-akustische-isolierungen/produktdetails/glasvlies-a/.

4 https://www.antratek.de/.

5 https://www.ics-schneider.de/.

6 https://www.infratec.de/.

7 https://www.muehlmeier.de/.

8 https://imagej.net/imaging/particle-analysis.

9 https://www.driesen-kern.de/.
