# Corrigendum to “Experimental dataset of fluid flow and heat transfer in a shallow packed bed at low Reynolds numbers” [Data in Brief 61 (2025) 111743]

**DOI:** 10.1016/j.dib.2026.113089

**Published:** 2026-07-16

**Authors:** Anika Weber, Sebastian Schürg, Timo Roeder, Johannes Grobbel, Martina Neises-von Puttkamer, Christian Sattler

**Affiliations:** a German Aerospace Center (DLR), Institute of Future Fuels, Im Langenbroich 13, 52428, Jülich, Germany; b German Aerospace Center (DLR), Institute of Future Fuels, Linder Höhe, 51147, Cologne, Germany; c RWTH Aachen University, Chair for Solar Fuel Production, Templergraben 55, Aachen, Germany

The authors would like to include in the Acknowledgements their thanks to **Hossam Amin**, former colleague of the DLR Institute of Future Fuels, for his valuable input and technical support during the set-up of the experimental test stand and the provision of the measurement systems.

The authors would like to apologise for any inconvenience caused.

